# CTISL: a dynamic stacking multi-class classification approach for identifying cell types from single-cell RNA-seq data

**DOI:** 10.1093/bioinformatics/btae063

**Published:** 2024-02-05

**Authors:** Xiao Wang, Ziyi Chai, Shaohua Li, Yan Liu, Chen Li, Yu Jiang, Quanzhong Liu

**Affiliations:** Department of Software Engineering, College of Information Engineering, Northwest A&F University, Yangling 712100, China; Department of Software Engineering, College of Information Engineering, Northwest A&F University, Yangling 712100, China; Department of Software Engineering, College of Information Engineering, Northwest A&F University, Yangling 712100, China; School of Computer Science and Engineering, Nanjing University of Science and Technology, Nanjing 210094, China; Department of Biochemistry and Molecular Biology, Monash University, Melbourne, VIC 3800, Australia; Department of Animal Genetics, Breeding and Reproduction, College of Animal Science and Technology, Northwest A&F University, Yangling 712100, China; Department of Software Engineering, College of Information Engineering, Northwest A&F University, Yangling 712100, China; Shaanxi Engineering Research Center of Agricultural Information Intelligent Perception and Analysis, Northwest A&F University, Yangling 712100, China

## Abstract

**Motivation:**

Effective identification of cell types is of critical importance in single-cell RNA-sequencing (scRNA-seq) data analysis. To date, many supervised machine learning-based predictors have been implemented to identify cell types from scRNA-seq datasets. Despite the technical advances of these state-of-the-art tools, most existing predictors were single classifiers, of which the performances can still be significantly improved. It is therefore highly desirable to employ the ensemble learning strategy to develop more accurate computational models for robust and comprehensive identification of cell types on scRNA-seq datasets.

**Results:**

We propose a two-layer stacking model, termed CTISL (Cell Type Identification by Stacking ensemble Learning), which integrates multiple classifiers to identify cell types. In the first layer, given a reference scRNA-seq dataset with known cell types, CTISL dynamically combines multiple cell-type-specific classifiers (i.e. support-vector machine and logistic regression) as the base learners to deliver the outcomes for the input of a meta-classifier in the second layer. We conducted a total of 24 benchmarking experiments on 17 human and mouse scRNA-seq datasets to evaluate and compare the prediction performance of CTISL and other state-of-the-art predictors. The experiment results demonstrate that CTISL achieves superior or competitive performance compared to these state-of-the-art approaches. We anticipate that CTISL can serve as a useful and reliable tool for cost-effective identification of cell types from scRNA-seq datasets.

**Availability and implementation:**

The webserver and source code are freely available at http://bigdata.biocie.cn/CTISLweb/home and https://zenodo.org/records/10568906, respectively.

## 1 Introduction

Single-cell RNA (scRNA)-seq techniques have been widely applied to profile transcriptomic data at the single-cell level (Tang *et al.* 2009). Identification of the cell types has therefore become a critical step in scRNA-seq data analysis (Huang and Zhang 2021). Traditionally, the types of cells were annotated based on their shapes, sizes, and other features observed via microscope anatomy, histology, and pathology (Arendt *et al.* 2016, Kiselev *et al.* 2019). The advances in scRNA-seq techniques have allowed the fast accumulation of sequencing data, which requires the assistance of computational and artificial intelligence-guided approaches for the accurate and robust annotation of cell types (Ma *et al.* 2021, Sun *et al.* 2022). Particularly, machine learning (ML)-based approaches have shown their great capability of handling large-scale datasets for annotating cell types. In recent years, various ML-based approaches have been developed to annotate cell types using scRNA-seq datasets. These methods can be broadly classified into two main categories: unsupervised and supervised approaches.

Unsupervised methods, such as RaceID (Grün *et al.* 2015), SNN-Cliq (Xu and Su 2015), SINCERA (Guo *et al.* 2015), pcaReduce (Žurauskienė and Yau 2016), GiniClust (Jiang *et al.* 2016), SIMLR (Wang *et al.* 2017), SC3 (Kiselev *et al.* 2017), SCENIC (Aibar *et al.* 2017), Scanpy (Wolf *et al.* 2018), and SCENA (Cui *et al.* 2021), cluster cells into different groups using various methods, e.g. *K*-means (Celebi *et al.* 2013), Shared Nearest Neighbor (Ertoz *et al.* 2002), and cluster-based similarity partitioning algorithm (Strehl and Ghosh 2003). Although unsupervised clustering-based methods achieved outstanding performances in annotating cell types, many challenges remain (Kiselev *et al.* 2019), such as the ambiguous number of cell-type clusters (Qi *et al.* 2020), model and parameter dependency (Xie *et al.* 2019), unsatisfactory performance on imbalanced clusters (Ma *et al.* 2021), and poor reproducibility (Chen *et al.* 2022). Moreover, unsupervised methods are usually constructed based on the assumption that all cells in each cluster belong to the same cell type. In fact, a cluster is often mixed with a major cell type and small percentages of other cell types (Alquicira-Hernandez *et al.* 2019), which makes it challenging to effectively annotate cell types in such clusters (Kiselev *et al.* 2019, Pliner *et al.* 2019).

Supervised ML-based methods, on the other hand, have been increasingly popular in recent years (Ma *et al.* 2021). These supervised methods can be further classified into two categories: traditional ML- and deep learning (DL)-based approaches. Traditional ML-based approaches, such as scPred (Alquicira-Hernandez *et al.* 2019), scClassify (Lin *et al.* 2020), ELSA (Wang *et al.* 2020a), scDetect (Shen *et al.* 2021), CellTypist (Domínguez Conde *et al.* 2022), scmap (Kiselev *et al.* 2018), SingleR (Aran *et al.* 2019), and scAnnotatR (Nguyen and Griss 2022), often employ classic ML models, such as Support-Vector Machines (SVM) (Cortes and Vapnik 1995), *K*-Nearest Neighbors (Cover and Hart 1967), Random Forest (RF) (Breiman 2001), Logistic Regression (LR) (Lecessie and van Houwelingen 1992), and Bootstrap (Fushiki 2010), etc. While DL-based approaches employ state-of-the-art neural network-based algorithms, including Artificial Neural Networks (Yang 2008), Convolutional Neural Networks (Yamashita *et al.* 2018), Deep Neural Networks (Samek *et al.* 2021), Capsule Neural Networks (Sabour *et al.* 2017), and Graph Neural Networks (Zhou *et al.* 2020), to achieve superior annotation performance. Representative DL-based approaches include SuperCT (Xie *et al.* 2019), ACTINN (Ma and Pellegrini 2020), scCapsNet (Wang *et al.* 2020b), scGraph (Yin *et al.* 2022), GraphCS (Zeng *et al.* 2022), TripletCell (Liu *et al.* 2023), scBERT (Yang *et al.* 2022), and scDeepInsight (Jia *et al.* 2023).

Some comprehensive reviews have thoroughly evaluated a number of supervised models for the identification of cell types (Abdelaal *et al.* 2019, Huang and Zhang 2021, Ma *et al.* 2021). Most supervised-based methods first used a scRNA-seq dataset with known cell types to train a multi-class classification model and then loaded the trained model to predict the type of each cell in a new scRNA-seq dataset. Despite that supervised methods generally outperform unsupervised cell clustering methods (Ma *et al.* 2021), most of these supervised methods are based on a single classifier. On the other hand, it has been widely accepted that ensemble multiple classifiers usually outperform the performance of a single learner (Zhou 2012). For example, scDetect (Shen *et al.* 2021) achieved outstanding cell-type identification performance by implementing multiple *k*-Top Scoring Pairs classifiers with a weighted majority voting strategy to identify cell types from scRNA-seq data.

In this study, we developed a two-layer dynamic ensemble learning model, termed CTISL (Cell Type Identification by Stacking ensemble Learning), for accurate cell-type prediction in scRNA-seq data. In CTISL, cell-type identification is regarded as a multi-class classification task. A variety of features were extracted to build multiple base learners for different cell-type categories. We selected the best-performing classifiers as base learners in CTISL according to our extensive experiments and a recent study evaluating the performance of various individual classifiers in cell-type identification using scRNA-seq datasets (Huang and Zhang 2021). Our extensive performance benchmarking experiments on scRNA-seq datasets of various species, tissues, batches, and protocols show that CTISL achieved outstanding performance and strong stability compared to state-of-the-art traditional ML and DL methods, demonstrating that the stacking ensemble learning technique is an effective approach to achieving more robust performance for cell-type annotation. Overall, our dynamic ensemble learning model provides a promising approach for accurately identifying cell types in scRNA-seq datasets, with the potential to advance biomedical research and clinical applications.

## 2 Materials and methods

### 2.1 Dataset collection

We collected in total 17 scRNA-seq datasets of two species (*Homo sapiens* and *Mus musculus*) from various tissues, batches, and protocols to train and evaluate CTISL and other benchmarked methods. Among these datasets, seven are derived from human peripheral blood mononuclear cell (PBMC) samples (Ding *et al.* 2020) and were used for inter- and intra-dataset evaluations. Several pairs of datasets with different batches were extracted from various cells, including human blood dendritic cells (Villani *et al.* 2017), namely Dendritic_batch1 and Dendritic_batch2, and mouse retinal bipolar cells (Shekhar *et al.* 2016), namely Retina(5)_batch1 and Retina(5)_batch2, and Retina(19)_batch1 and Retina(19)_batch2. Each of the three pairs was used for cross-batch evaluations. In addition, two datasets from human and mouse airway (Plasschaert *et al.* 2018) and pancreas (Baron *et al.* 2016) were extracted, namely “HumanAirway,” “MouseAirway,” “HumanPancreas,” and “MousePancreas,” respectively. Each pair of datasets is from the same tissue of two species and was used for cross-species evaluations. A detailed description of these 17 scRNA-seq datasets is provided in Supplementary Table S1.

### 2.2 The CTISL framework

**Figure 1.**
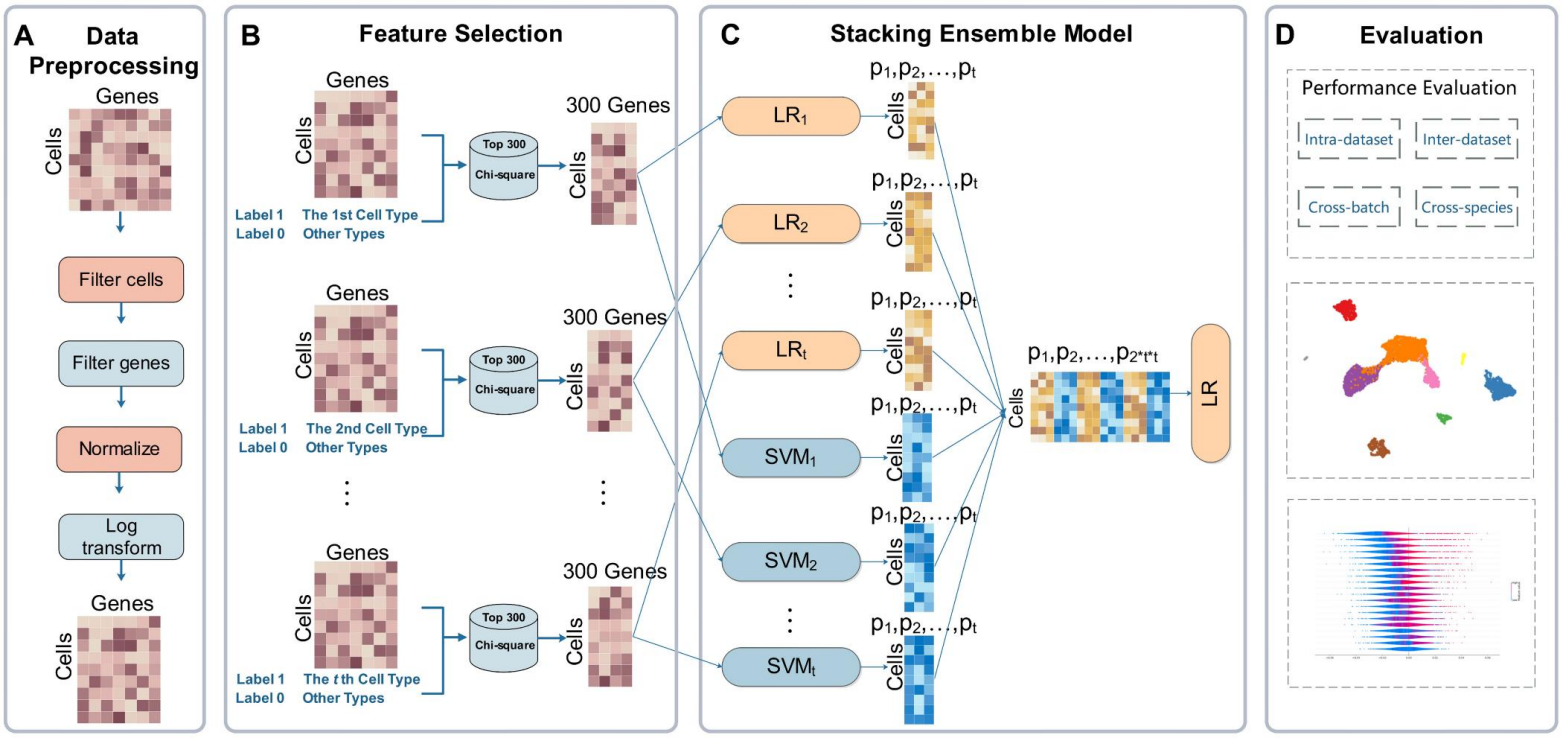
The construction of the CTISL framework includes four major steps, including (A) dataset pre-processing, (B) feature selection, (C) dynamic stacking model construction, and (D) model evaluation.

As illustrated in Fig. 1, the construction of CTISL consists of four steps: dataset pre-processing, feature selection, stacking model construction, and model evaluation.

**Step 1. Dataset pre-processing:** We employed the Scanpy package (Wolf *et al.* 2018) to perform scRNA-seq data pre-processing (Fig. 1A). In the cell normalization step, the expression value of each gene in each cell was divided by the total sum of gene expression values in that cell and then multiplied by a constant 10e4. Other pre-processing steps remain consistent with the previous study (Hu *et al.* 2020). Note that the dataset pre-processing step is performed separately for training and testing datasets.

**Step 2. Feature selection:** A scRNA-seq dataset D can be represented as an n×m matrix, where n and m are the numbers of cells and genes, respectively. $D_{i}\left(1\leq i\leq n\right)$ represents the *i*-th cell, and $D_{ij}\left(1\leq i\leq n,\;1\leq j\leq n\right)$ denotes the expression value of the j-th gene in $D_{i}$. As there is usually more than one cell type in D, cell-type identification is formulated as a multi-class classification task. Generally, D contains high-dimensional features (genes) and small size of samples (cells). The high number of redundant genes might decrease the predictive performance of the model. To find a better gene subset for identifying cell types, we employed χ^2^ (Forman 2003), a popular feature selection method, to select the genes related to cell types. Suppose D contains *t* cell types, and C represents the set of cell types, the procedure of feature selection (Fig. 1B) is described as follows: (i) a dataset ${D^{\prime }}_{k}$ with two classes was constructed for each cell type $C_{k}\;\left(1\leq k\leq t\right)$—samples with the cell type of $C_{k}$ are regarded as the positive class of ${D^{\prime }}_{k}$, and remaining samples as the negative class; and (ii) χ^2^ was employed to select the top 300 genes with stronger identification ability of $C_{k}$, and let $f_{k}$ denote the set of these 300 genes. We then repeated the above two steps until each $f_{k}\left(1\leq k\leq t\right)$ was obtained as informative genes for the cell type of $C_{k}$.

**Step 3. Stacking model construction:** Among traditional ML-based methods, SVM with the radial basis function (RBF) or linear kernel, and LR classifiers achieved generally better performances than other classifiers in annotating cell types (Abdelaal *et al.* 2019, Alquicira-Hernandez *et al.* 2019, Huang and Zhang 2021, Ma *et al.* 2021). In this work, we employed the stacking strategy (Zhou 2012) to integrate SVM and LR classifiers as individual base learners. It has been demonstrated that the stacking approach can achieve powerful performance in various bioinformatic tasks, such as anti-cancer peptide identification (Liang *et al.* 2021), prokaryotic lysine acetylation site prediction (Basith *et al.* 2022), and long ncRNA subcellular localization prediction (Cao *et al.* 2018). In addition, to conduct a more comprehensive study, we integrated another two base learners, including RF (Breiman 2001) and gradient boosting classifier (GBC) (Friedman 2001). We also built other variations for our CTISL framework, including χ^2^ with multilayer perceptron (Popescu *et al.* 2009) (χ^2^+MLP) and CTISL with marker genes. Refer to Supplementary Section S1 for more information. In this work, we constructed two layers of stacking ensemble models. The first layer combines SVM and LR as the base classifiers via the stacking strategy and the second level employs LR as the meta-classifier fed by the outputs of the stacking layer. Details of the procedure are described as follows (Fig. 1C). In the first step, given a scRNA-seq dataset D with n cells and the set C with *t* kinds of cell types, we obtained a set of informative genes $f_{k}\left(1\leq k\leq t\right)\;$for the cell type of $C_{k}\left(1\leq k\leq t\right)\;$using the proposed feature selection strategy. Subsequently, we extracted these gene columns in $f_{k}$ and removed other genes from D to form a sub-dataset $D\left(f_{k}\right)$. In the second step, to avoid overfitting, we implemented our ensemble strategy using the stacking cross-validation algorithm provided in the “mlxtend” package (Raschka 2018). In this procedure, $D\left(f_{k}\right)\;$was first split into 3-folds. The first-level classifier SVM with RBF kernel was trained on the 2-folds, and prediction results (t probability values) on the remaining fold as new features of the fold. After three rounds, $D\left(f_{k}\right)\;$was transformed into a new dataset $D'\left(f_{k}\right)\;$with t features. The same procedure was used to fit the first layer LR classifier. Thus, $D\left(f_{k}\right)\;$was transformed into a new dataset $D'\left(f_{k}\right)\;$with 2t features through the first layer classifiers. After repeating the above two steps for each $D\left(f_{k}\right),\left(1\leq k\leq t\right)$, D was transformed into a new dataset $D\prime =U_{k=1}^{t}\;D'\left(f_{k}\right)$, where D' is an n×(t×2t) matrix. Finally, D' was fed into the second layer classifier LR. Note that as the numbers of cell types vary across different scRNA-seq datasets, CTISL dynamically combines different numbers of base classifiers (i.e. SVM and LR) on different scRNA-seq training datasets.

**Step 4. Model evaluation:** To effectively evaluate and compare the performance of CTISL and other state-of-the-art approaches, we used four evaluation strategies including intra-dataset, inter-dataset, cross-batch, and cross-species evaluation (Fig. 1D). For intra-dataset validation, we used the 5-fold cross-validation on seven PBMCs of *H.sapiens*. Five-folds were stratified to preserve the ratios of samples/cells for each cell type. When validating the inter-dataset performance, we used seven human PBMC datasets, which were generated by seven different protocols (i.e. 10Xv2, 10Xv3, CEL-Seq2, Drop-Seq, inDrop, SMART-Seq2, and SeqWell, respectively). To maximize the use of the existing datasets, we conducted seven inter-group experiments, with each experiment using one dataset as the testing set and the remaining six datasets for training. For cross-batch validation, we used three datasets, Dendritic, Retina(5), and Retina(19). For each dataset, we conducted experiments with one batch for training and the other one for testing. In total, we conducted six cross-batch experiments. To assess the cross-species performance, we conducted experiments on a pair of datasets, which were obtained from the same tissue of two different species by the same protocol. The model was trained on one dataset of original species and predicted cell types in another dataset of target species, and *vice versa*. In this work, we used two datasets generated by the inDrop protocol from the pancreas tissues of *H.sapiens* and *M.musculus* and two datasets generated by the inDrop protocol from the airway tissues of *H.sapiens* and *M.musculus*.

### 2.3 Benchmarking against state-of-the-art ML-based cell-type prediction methods

In our benchmark experiments, we compared CTISL with nine state-of-the-art ML-based methods including ACTINN (Ma and Pellegrini 2020) scCapsNet (Wang *et al.* 2020b), scDetect (Shen *et al.* 2021), TripletCell (Liu *et al.* 2023), scmap-cluster (Kiselev *et al.* 2018), scmap-cell (Kiselev *et al.* 2018), CellTypist (Domínguez Conde *et al.* 2022), scBERT (Yang *et al.* 2022), and SingleR (Aran *et al.* 2019). For ACTINN, scDetect, TripletCell, scmap-cluster, scmap-cell, CellTypist, scBERT, and SingleR, we used the pre-processing methods provided in their studies to process the original data and used the processed data as input for these models. While for scCapsNet, we used the method in this study to pre-process the data. All compared methods were trained and tested using the same training and testing datasets to ensure a fair comparison. We employed three popular performance evaluation metrics, including accuracy, median *F*1-score, and macro *F*1-score (Supplementary Section S2) to evaluate and compare the predictive performance of CTISL with state-of-the-art approaches. Accuracy is defined as the percentage of correctly predicted cell type among all cells. Median *F*1-score is defined as the median value of *F*1-scores of all cell types and macro *F*1-score denotes the average of *F*1-scores of all cell types. Therefore, macro *F*1-score is suitable for scRNA-seq data with highly imbalanced proportions of cell types (Ma *et al.* 2021).

## 3 Results

### 3.1 Performance evaluation on selected feature genes

**Figure 2.**
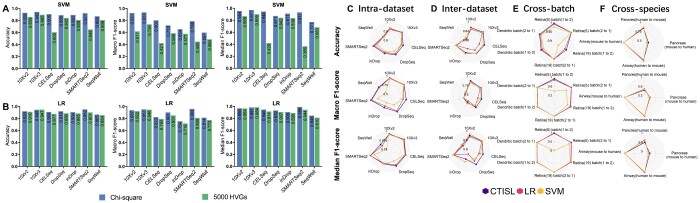
Feature gene selection and performance comparison of base learners and our CTISL framework (stacking LR and SVM). (A and B) Performance of SVM and LR using different feature gene selection approaches including the χ^2^ method (300 genes selected) and the 5000 highly variable genes used in the work by Huang and Zhang (2021). (C–F) Performance comparison among base classifiers LR, SVM, and CTISL (using the stacking ensemble learning technique) models based on the 300 selected feature genes by the χ^2^ method in terms of accuracy, macro *F*1-score, and median *F*1-score using (C) intra-dataset, (D) inter-dataset, (E) cross-batch, and (F) cross-species evaluation strategy.

Most feature gene selection algorithms were designed to choose highly variable genes (HVGs). However, cell-type identification is a multi-class classification task and HVGs might not be related to cell types. This section therefore aimed to evaluate the performance of the base classifiers on different feature gene sets, including feature genes selected by various methods and HVGs. The feature gene selection methods, we evaluated in this study include the χ^2^ method, limma (Ritchie *et al.* 2015), and GeneClust (Deng *et al.* 2023). While the χ^2^ method and limma can select feature genes for each cell type, GeneClust selects a subset of highly representative genes that are relevant to each cluster. For each cell type, we used the χ^2^ algorithm and limma to select the top *k* (*k* = 100, 200, 300, 400, and 500) genes that are related to cell types. Thus, for scRNA-seq with *t* cell types, t×k genes were chosen as features. To evaluate the performance of genes selected by our proposed feature selection method, we compared the selected genes in our work with 2000, 3000, 4000, and 5000 highly variable genes, respectively. We trained our base classifiers on seven human scRNA-seq datasets using the selected genes via a 5-fold cross-validation test—the feature genes were selected using the 4-folds and the performance was tested using the rest fold. Performance comparison between SVM and LR using 300 χ^2^-selected genes and 5000 HVGs is shown in Fig. 2A and B, respectively. Detailed performance values of all the numbers of selected genes and feature gene selection methods are shown in Supplementary Tables S2 and S3, respectively. From the average accuracy, and macro and media *F*1-scores, the χ^2^ method outperformed other gene selection methods and HVGs. However, the performance did not always improve with the increase in the number of features selected by the χ^2^ method. Therefore, we further evaluated the performance of the whole CTISL framework on various numbers of selected genes (from 10 to 2000) selected by the χ^2^ approach for all experimental scenarios, including intra-dataset, inter-dataset, cross-batch dataset, and cross-species. As illustrated in Supplementary Fig. S1, not all performance values generally improved with the increase in the number of selected genes, posing the challenge of selecting a universally optimized number of genes. Specifically, we counted the number of times when each number of selected genes achieved the highest accuracy and macro and median *F*1-scores. As a result, 2000 χ^2^-selected genes achieved the highest performance 20 times, followed by 1500 genes (18 times) and 300 genes (16 times). However, when using the selected 2000 and 1500 genes, CTISL took a significantly longer time to build the model. Considering balancing the running time and performance, in our CTISL framework, we used 300 as the default number of top genes based on the experiments. We further used the 10Xv2 dataset as an example to explore the robustness of CTISL concerning different numbers of distinct genes selected for each cell type using the χ^2^ approach (Supplementary Table S4 and Supplementary Fig. S2). Notably, we observed a reduction in the number of distinct genes for certain cell types with an increase in the number of selected genes. Moreover, when the number of selected genes was above 1000, the number of distinct signature genes for each cell type did not always increase along with the increase of the number of selected genes by the χ^2^ approach. Refer to Supplementary Section S1 for more details.

We also examined the genes selected by the χ^2^ method and found that some of them are marker genes of the corresponding cell types according to the CellMarker 2.0 database (Hu *et al.* 2023). As shown in Supplementary Table S5, 31 out of 62 marker genes in B cells of the PBMC_10Xv2 dataset were selected by the χ^2^ method. These findings confirm that the χ^2^ method is capable of selecting indicative feature genes for cell-type identification. We then conducted a performance comparison of CTISL by using selected feature genes and marker genes, respectively. We found that CTISL achieved better performance when using the selected feature genes in comparison to the marker genes (Supplementary Table S6). Although maker genes have a strong ability to identify cell types, the limited number of known marker genes in some cell types cannot help the model achieve satisfactory performance of cell-type identification. Refer to Supplementary Section S2 for more information.

### 3.2 Performance evaluation of CTISL

We then evaluated and compared the performance improvement achieved by the stacking learning technique. We systematically compared CTISL (using stacking learning to ensemble LR and SVM) with selected genes and marker genes, respectively, χ^2^+MLP (multilayer perceptron) (Popescu *et al.* 2009), and CTISL with LR+SVM+RF+GBC (Friedman 2001) in intra-dataset experiments, inter-dataset experiments, cross-batch experiments, and cross-species experiments. Refer to the methodological details of integrating MLP, RF, and GBC in Supplementary Sections S1 and S3. The performance of CTISL stacking LR and SVM using 300 selected feature genes is demonstrated in Fig. 2 and all detailed performance values are illustrated in Supplementary Table S6.

The intra-dataset experiments demonstrate that CTISL outperformed all other approaches in terms of average accuracy and average median *F*1-score, except for the slightly lower (0.001) average macro *F*1-score than CTISL with LR+SVM+RF+GBC (Supplementary Table S6). In addition, CTISL outperformed the base classifiers LR and SVM (Fig. 2C and Supplementary Table S6) in terms of average accuracy (92.8% versus 91.6% and 92.8% versus 90.7%), average macro *F*1-score (0.906 versus 0.877 and 0.906 versus 0.773), and average median *F*1-score (0.947 versus 0.938 and 0.947 versus 0.908). While for inter-dataset experiments, compared to LR and SVM, CTISL achieved higher average accuracy (87.2% versus 83.0% and 87.2% versus 87.1%), average macro *F*1-score (0.734 versus 0.696 and 0.734 versus 0.716, respectively), and average median *F*1-score (0.857 versus 0.801 and 0.857 versus 0.850) in inter-dataset experiments (Fig. 2D and Supplementary Table S6). For cross-batch experiments, CTISL outperformed the base classifiers LR and SVM (Fig. 2E and Supplementary Table S6) in terms of average accuracy (98.3% versus 97.8% and 98.3% versus 96.6%), average macro *F*1-score (0.961 versus 0.948 and 0.961 versus 0.798), and average median *F*1-score (0.973 versus 0.971 and 0.973 versus 0.778). Our cross-species experiments demonstrate that χ^2^+MLP outperformed CTISL in average macro and median *F*1-scores, while CTISL still achieved better performance than the base learners (i.e. LR and SVM) in most of the cross-species experiments (Fig. 2F and Supplementary Table S6). Detailed performance comparison and discussion about χ^2^+MLP and CTISL with LR+SVM+RF+GBC have been provided in Supplementary Section S3.

**Figure 3.**
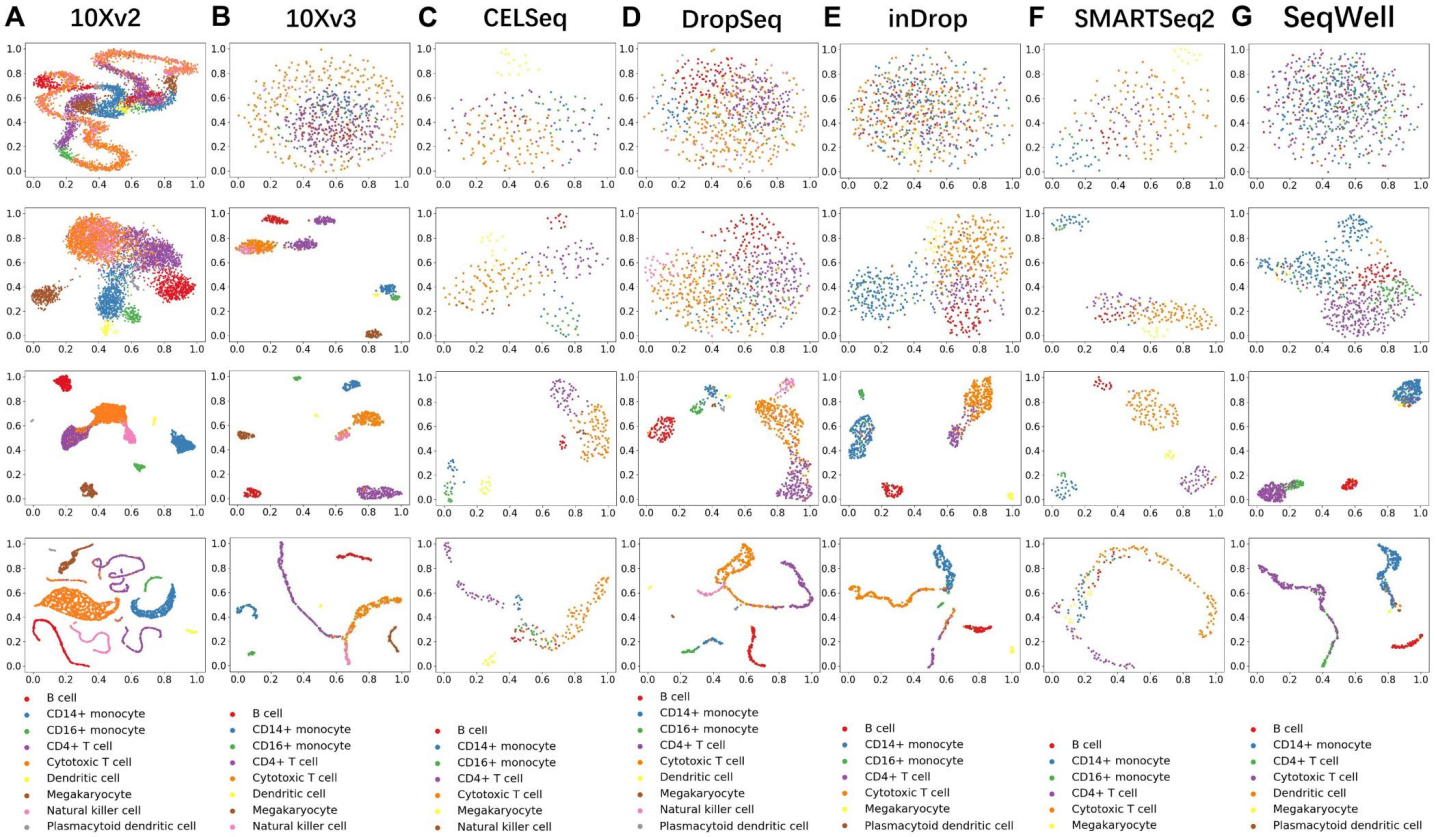
Visualizing the cell-type identification results on seven datasets, including (A) 10Xv2, (B) 10Xv3, (C) CELSeq, (D) DropSeq, (E) inDrop, (F) SMARTSeq2, and (G) SeqWell. For each dataset, the four panels, from top to bottom, represent the visualizations without feature selection, the results after feature selection, the output of the first layer of the model, and the output of the last layer of the model, respectively.

Although CTISL did not outperform the ensemble base learners on all three metrics in all 24 benchmarking experiments, the results demonstrate that CTISL achieved consistently robust and stable average performance across all cases, except for the comparative prediction performance based on cross-species. Overall, we conclude that the stacking learning technique can effectively improve the prediction performance of base classifiers on intra-dataset, inter-dataset, and cross-batch scenarios of cell type-identification. In addition, to examine the effectiveness of CTISL, we employed UMAP (McInnes *et al.* 2018) to visualize the cell types represented by features extracted from each step in our model (Fig. 3). As can be seen from Fig. 3, all cells were mixed in the original dataset. After selecting the feature genes, the same type of cells began to form a blurry cluster, which then gradually separated after the first layer. After the output layer, the same type of cells was well clustered into the same group. These results confirm that CTISL can effectively extract informative features representing cell types, thereby achieving outstanding prediction performance.

### 3.3 Model interpretation

In this section, we used the SHAP package (Lundberg and Lee 2017) to interpret the output of CTISL (Supplementary Fig. S3) and compared the selected feature genes with the marker genes from the CellMarker 2.0 database (Hu *et al.* 2023). We plotted the distribution of the impact of the top 20 selected genes from the output of CTISL. Positive SHAP values indicate the identification of the current cell type, while negative SHAP values indicate the identification of other types. Taking the B cell in 10Xv2 e.g. (Supplementary Table S5 and Supplementary Fig. S3A), most genes with high expression achieved positive SHAP values. Among these 20 selected top genes, 8 are marker genes according to CellMarker 2.0, including CD79A, IGHM, MS4A1, TNFRSF13C, IGHD, IGKC, CD74, and CD79B. These genes have high expression values in most B cell samples, and their SHAP values from the output of the model are positive, meaning that these genes are favorable to the identification of B cells. Similar results of other cell types can also be found in 10Xv2 (Supplementary Fig. S3). For example, the high expression of IL7R and LTB are marker genes of CD4+ T cells according to CellMarker 2.0 and are favorable for the recognition of CD4+ T cells (Supplementary Fig. S3I). They were among the top 20 selected genes for this cell type according to the output of our CTISL. Similarly, the high expression of PF4, PPBP, TUBB1, and MYL9 positively impacted the identification of megakaryocytes (Supplementary Fig. S3F), and they were marker genes for this cell type based on our SHAP analysis. In addition, high expression of marker genes, such as KLRF1, SPON2, KLRB1, GNLY, CCL4, FCGR3A, CD247, GZMB, CD7, KLRD1, and TRDC are more indicative of identifying natural killer cells. These genes are all marker genes based on the annotations of CellMarker 2.0 and were also among the top 20 selected genes for this cell type based on the outputs of CTISL (Supplementary Fig. S3G). For the Plasmacytoid dendritic cell (Supplementary Fig. S3H), out of the 6188 samples in the PBMC-10Xv2 dataset, only 38 samples are available. As such, there are only eight plasmacytoid dendritic cells in the test sub-dataset using a 5-fold cross-validation test on the 10Xv2 dataset. As a result, there are few data points with higher SHAP values depicted in Supplementary Fig. S3H.

### 3.4 Benchmarking CTISL against state-of-the-art methods in intra- and inter-dataset scenarios

**Figure 4.**
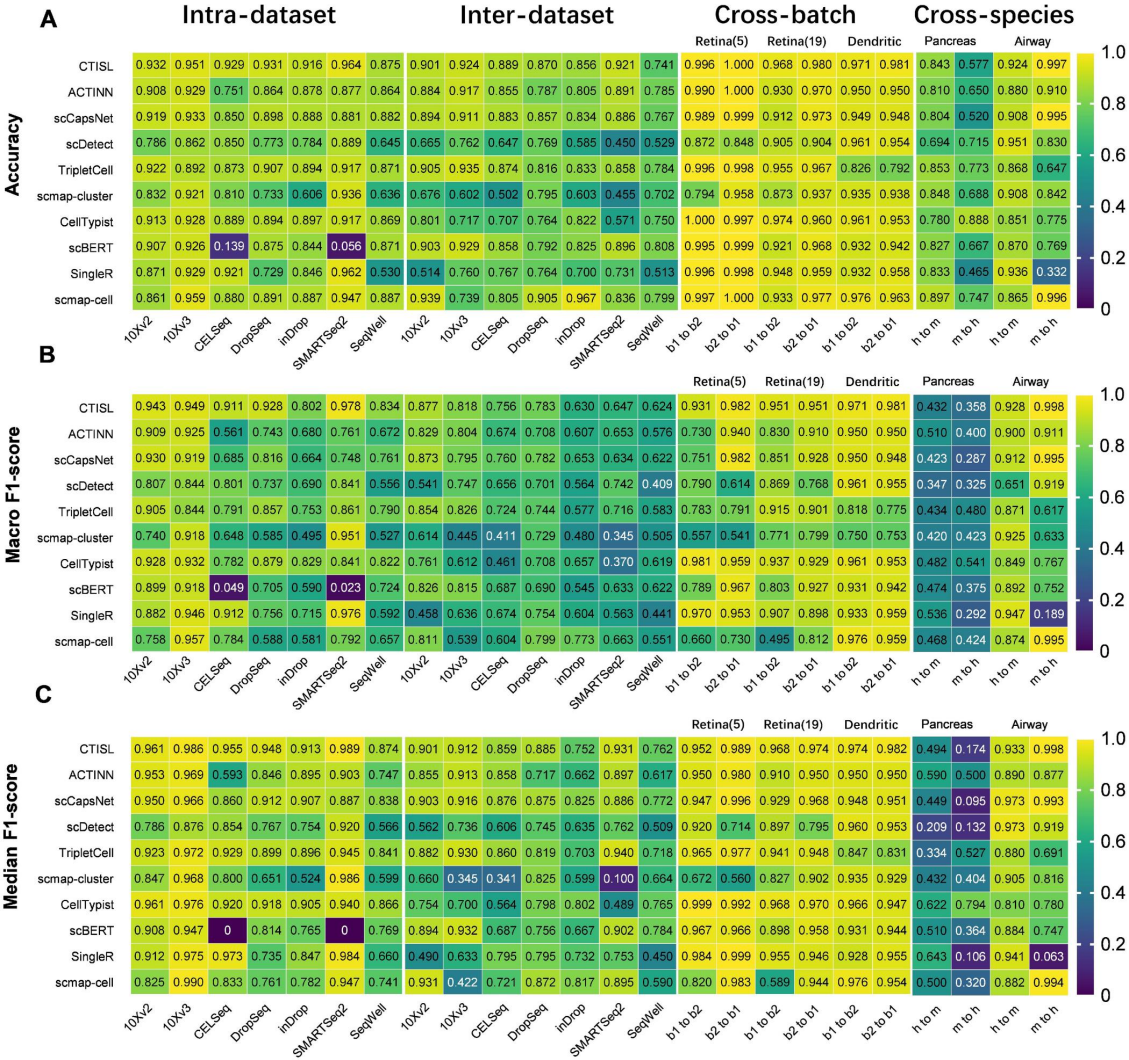
Performance evaluation and benchmarking between CTISL and nine state-of-the-art cell-type predictors in terms of (A) accuracy, (B) macro *F*1-score, and (C) median *F*1-score on seven human PBMC datasets in intra- and inter-dataset, cross-batch, and cross-species experiments. “b1 to b2” and “b2 to b1” mean that the model was trained on the first/second batch of dataset and tested on the second/first batch of dataset, respectively. While “h to m” and “m or h” mean that the model was trained on the human/mouse dataset and was tested using the mouse/human dataset, respectively.

We first performed 5-fold cross-validations on seven human PBMC datasets and compared CTISL with nine state-of-the-art approaches in intra- and inter-dataset scenarios. The evaluated approaches included ACTINN, scCapsNet, scDetect, TripletCell, scmap-cluster, scmap-cell, CellTypist, scBERT, and SingleR. Figure 4A demonstrated that CTISL outperformed all other nine benchmarked methods in terms of accuracy on five out of seven intra-dataset experiments. Among the remaining two datasets, scmap-cell achieved the highest accuracy of 95.9%, while CTISL achieved the second-best performance of 95.1% on the 10Xv3 dataset. Additionally, CTISL achieved the third-best performance of 87.5% on the SeqWell dataset. In terms of macro *F*1-score (Fig. 4B), CTISL outperformed all other nine benchmarked methods on four out of seven intra-datasets. For the remaining three datasets, CTISL achieved the second-best performance of 0.949 on the 10Xv3 dataset, 0.911 on the CELSeq dataset, and 0.802 on the inDrop dataset. Similar results are observed in terms of median *F*1-score (Fig. 4C). To evaluate the performance generalization of CTISL, we further performed inter-dataset tests on seven human PBMC datasets generated by different protocols. We first trained our model on six of seven datasets and tested the model on the remaining dataset. Each dataset was used as the testing data once [in line with Wang *et al.* (2020b) and Yang *et al.* (2022)] and the evaluation therefore contained seven sub-tasks. As can be seen in Fig. 4A, CTISL achieved the best accuracy on two out of seven tests. In the remaining five datasets, CTISL achieved the second-best performance of 87.0% on the DropSeq test dataset, the second-best performance of 85.6% on the inDrop test dataset, the fourth-best performance of 90.1% on the 10Xv2 test dataset, the third-best performance of 92.4% on the 10Xv3 test dataset, and the seventh-best performance of 74.1% on the SeqWell test dataset. Additionally, CTISL and scmap-cell achieved the highest macro *F*1-score on two out of seven test results (Fig. 4B), followed by scCapsNet, scDetect, and TripletCell on one test, respectively. scCapsNet, and scBERT achieved the highest median *F*1-score on two tests, followed by CTISL, TripletCell, and scmap-cell on one test separately (Fig. 4C). Overall, these comparison results demonstrate that CTISL is capable of accurately and robustly identifying cell types in both inter- and intra-dataset experiments.

### 3.5 Performance comparison in cross-batch and cross-species scenarios

To evaluate the performance of CTISL on different batches of datasets, we ran all 10 predictors on 3 datasets with 2 batches (Supplementary Table S1). We trained all models on one batch and assessed their performance using the dataset of another batch. In terms of accuracy (Fig. 4A), CTISL, ACTINN, and scmap-cell achieved the highest accuracy (100%) across one batch of the Retina(5) dataset (b2–b1). Similarly, CTISL achieved the highest performance of 98.0% on the Retina(19) dataset (b2–b1) and the second-best performance of 96.8% (b1–b2). Similarly, CTISL achieved the highest performance of 98.1% on the dendritic dataset (b2–b1) and the second-best performance of 97.1% (b1–b2). CTISL also achieved the highest macro and median *F*1-scores (Fig. 4B and C) on four and three out of six experiments, respectively.

We then conducted cross-species cell-type identification using human and mouse pancreas and airway datasets. CTISL achieved the fourth-best accuracy of 84.3% from human to mouse on the pancreas datasets (Fig. 4A). Except for CellTypist, which achieved the highest accuracy of 88.8%, all other models performed poorly when they were trained on the mouse pancreas dataset to predict cell types in the human pancreas dataset. This is presumably because those cell types are not completely identical across human and mouse pancreas tissues. It is a common phenomenon in cross-species scenarios that some unique cell types only exist in the test dataset. For example, several cell types (e.g. acinar, epsilon, and mast) only appear in the human pancreas dataset and are not present in the mouse pancreas dataset. Similarly, two cell types (e.g. B_cell and immuse_other) solely appear in the mouse pancreas dataset and are not present in the human pancreas dataset (Supplementary Table S7). This phenomenon greatly reduces the prediction performance of all the models compared. On the other hand, in the airway dataset, the human data comprised three cell types—Basal, Ciliated, and Secretory, with similar sample sizes of 252, 258, and 280, respectively. When using human airway datasets for training the model to predict the cell types in mouse airway datasets, all 10 models performed well, with CTISL ranked third in accuracy (Fig. 4A). However, the mouse airway datasets exhibited a huge imbalance in sample sizes of the cell types, including Basal, Ciliated, and Secretory, with sample sizes of 6009, 1333, and 4792, respectively. Therefore, we applied the “RandomUnderSampler” function from the “imblearn” library (Lemaître *et al.* 2017), which is a down-sampling strategy based on the method proposed by Laurikkala (2001) to balance the class distribution in the dataset (Supplementary Section S3). We set the sample size of each cell type to 1333 and trained the four models using the down-sampled data to predict human data. Five rounds of down-sampling were then conducted for each experiment and the average results for each trial were calculated. CTISL achieved the highest accuracy of 99.7% (Fig. 4A) and the highest macro (Fig. 4B) and median *F*1-score (Fig. 4C) of 0.998 on the human airway dataset. In addition, according to cross-species experiments (Supplementary Table S6), χ^2^+MLP achieved overall better performance than CTISL, and CTISL with LR+SVM+RF+GBC outperformed CTISL in terms of average macro and median *F*1-score. Therefore, we recommend χ^2^+MLP when performing cross-species cell-type identification. Taken together, despite that χ^2^+MLP performed better than CTISL in cross-species experiments, these results showed that CTISL possesses a strong predictive ability in cell-type identification in intra-dataset, inter-dataset, and cross-batch experiment scenarios.

## 4 Discussion and conclusions

For identifying cell types using highly sparse single-cell expression matrix data, it is crucial to perform feature selection prior to model construction. Our feature selection method consists of multiple iterations, with each iteration selecting representative features for a specific cell type. We compared our selected features with publicly available cell-type marker genes and found that some of our selected marker genes could accurately identify the corresponding cell types and have significant biological relevance. Furthermore, we analyzed the performance of CTISL using various feature gene selection methods and different numbers of selected genes (Supplementary Tables S2 and S3, and Supplementary Section S1). It is worth noticing that the prediction performance of CTISL is still limited by supervised learning. When training and testing CTISL across datasets, the cell types in the predicted results of CTISL are solely based on the available types in the training set, meaning that the model cannot identify new cell types in the test dataset. Although some existing models are able to identify new cell types (such as ACTINN, scCapsNet, scDetect, TripletCell, scmap-cell, and scmap-cluster), they may only classify them as a “novel”/ “other” type without accurately determining their actual types. In the future, we will endeavor to incorporate more cell types by combining more single-cell omic data, such as scATAC-seq data, thereby enriching the prediction capacity of CTISL. CTISL utilizes LR and SVM as default base learners for each cell type in the training dataset. However, other classifiers can also be integrated into CTISL as base learners (Supplementary Sections S3). Moreover, as the number of cell types increases, CTISL can dynamically increase the number of base learners (Supplementary Section S5). In addition, the performance of CTISL, like most state-of-the-art predictors, may suffer from imbalanced cell types in the training dataset when performing cross-species predictions. However, this issue can be to some extent mitigated by employing down-sampling techniques in the training dataset. It is worth noticing that the down-sampling strategy does not work if the rarest cell type (i.e. the cell type with the least number of cells compared to other cell types) in the training dataset has an insufficient number of cells. We also discussed the effect of cell-type imbalances on different experiment scenarios, such as cross-batch and intra-dataset in Supplementary Section S4.

We developed a user-friendly web-based application for CTISL at http://bigdata.biocie.cn/CTISLweb/home to facilitate community-wide efforts to identify cell types using users’ datasets. Additionally, users have the option to choose different models other than LR and SVM as base learners or add additional models to the CTISL framework, via our webserver and locally runnable software (https://zenodo.org/records/10568906). Alternatively, given that χ^2^+MLP achieved better performance than the original CTISL in cross-species cell-type identification, we provided the MLP option on our webserver and GitHub repository for users to replace the stacking model. In conclusion, as a dynamic stacking ensemble learning-based model for robust multi-class classification of cell types using scRNA-seq data, CTISL achieved better or competitive, and more robust performances compared to other state-of-the-art predictors based on our extensive benchmarking experiments on intra- and inter-dataset, cross-batch, and cross-species datasets. Altogether, we anticipate that CTISL will serve as a prominent computational tool for the accurate identification of cell types using scRNA-seq data, thereby facilitating scRNA-seq data analysis and hypothesis generation.

## Supplementary Material

btae063_Supplementary_Data
