# Supplementary material for: CTISL: a dynamic stacking multi-class classification approach for identifying cell types from single-cell RNA-seq data
Source: Bioinformatics. 2024 Feb 5;40(2):btae063. doi: 10.1093/bioinformatics/btae063 (PMC10873586; doi:10.1093/bioinformatics/btae063)
Supplement: btae063_Supplementary_Data [file btae063_supplementary_data.docx]

**CTISL: a dynamic stacking multi-class classification approach for identifying cell types from single-cell RNA-sequencing data**

Xiao Wang^1^, Ziyi Chai^1^, Shaohua Li^1^, Yan Liu^2^, Chen Li^3,*^, Yu Jiang^4,*^, Quanzhong Liu^1,5,*^

^1^Department of Software Engineering, College of Information Engineering, Northwest A&F University, Yangling 712100, China; ^2^School of Computer Science and Engineering, Nanjing University of Science and Technology, 200 Xiaolingwei, Nanjing, 210094, China; ^3^Department of Biochemistry and Molecular Biology, Monash University, Melbourne, VIC 3800, Australia; ^4^Department of Animal Genetics, Breeding and Reproduction, College of Animal Science and Technology, Northwest A&F University, Yangling 712100, China; ^5^Shaanxi Engineering Research Center of Agricultural Information Intelligent Perception and Analysis

^*^To whom correspondence should be addressed: [chen.li@monash.edu](mailto:chen.li@monash.edu), [liuqzhong@nwafu.edu.cn](mailto:liuqzhong@nwafu.edu.cn), and [yu.jiang@nwafu.edu.cn](mailto:yu.jiang@nwafu.edu.cn).

**Supplementary Methods**

**1. Variations of the CTISL framework**

Besides our classic CTISL model (stacking LR and SVM), we also built and evaluated some variations of our CTISL framework, including CTISL with marker genes, Chi-square+MLP, and CTISL with LR+SVM+RF+GBC.

*CTISL with marker genes*: We used the marker genes specific to each cell type in the training dataset to replace the top 300 feature genes selected by the Chi-square method for each cell type. LR and SVM were stacked in the CTISL model.

*Chi-square+MLP*: For each cell type, we used the Chi-square approach to select feature genes (**Figure 1B**). These genes were then used as the input for the MLP (multilayer perceptron) model (Popescu, et al., 2009), which was implemented using two fully connected layers and an output layer with a softmax activation function.

*CTISL with LR+SVM+RF+GBC*: The base learners (**Figure 1C**) within the CTISL stacking framework include LR, SVM, RF (Random Forest) (Breiman, 2001), and GBC (Gradient Boosting Classifier) (Friedman, 2001).

**2. Evaluation Metrics**

To quantify and compare the performance of CTISL with other methods, three common performance evaluation metrics were used, including accuracy, macro F1-score, and median F1-score. The detailed definitions of these measures are as follows:

$$accuracy=\frac{Number of correct predictions}{All predictions},$$

$${F1}_{i}=\frac{2\times{TP}_{i}}{2\times{TP}_{i}+{FP}_{i}+{FN}_{i}},$$

$$Macro F1=\frac{\sum_{i=1}^{n} {F1}_{i}}{n},$$

$$Median F1=Median\left( {F1}_{1},{F1}_{2,}\cdots,{F1}_{n} \right),$$

where *TP_i_*, *FP_i_*, *TN_i_*, and *FN_i_* represent the number of true positives, true negatives, false positives, and false negatives, respectively, for the current cell type *i*.

**3. The down-sampling strategy**

Given a scRNA-seq dataset $D$ consisting of *t* cell types, let $C$ be the set that includes *t* types of cells, and $C_{i}\left( 1\leq i\leq t \right)$ denote the *i*-th cell type. When the proportions of cell types are highly imbalanced in $D$, we implemented a down-sampling strategy to construct a balanced dataset. This strategy was applied using the ‘RandomUnderSampler’ function from the ‘imblearn’ library (Lemaître, et al., 2017), which is a downsampling strategy based on the method proposed by Laurikkala (Laurikkala, 2001). We constructed a balanced dataset in the following steps:

Step 1: We initialized a balanced dataset $D'$ as all cell samples with a cell type of $C_{k}\left( 1\leq k\leq t \right)$ which has the smallest size of samples in $D$.

Step 2: For each other cell type $C_{j}\left( 1\leq j\leq t,j\neq k \right)$, we randomly extracted $\left| C_{k} \right|$ samples with the cell type of $C_{j}$ from $D$, and then put them into $D'$.

Step 3: We repeated Step 2 until samples from all other cell types were extracted.

To avoid the bias caused by a single down-sampling, we conducted the down-sampling multiple times, resulting in multiple balanced datasets. The tested model was then evaluated on each of these multiple balanced datasets and the average performance on multiple datasets was reported as the final results.

**Supplementary Results and Discussion**

- - - 1. **The robustness of CTISL concerning different numbers of genes selected using the Chi-square approach**

We conducted evaluations of CTISL on various numbers of selected genes by the Chi-square-based approach. As illustrated in **Figure S1**, not all performance values generally increased as the number of selected genes increased, posing the challenge of selecting a universally optimized number of genes. Specifically, the accuracies demonstrated an upward trend as the dimension increased when it was below 300. However, when the number of genes was above 300, the performance did not significantly improve in most scenarios as the number of selected genes increased. This indicates that the addition of more genes did not always enhance the performance of CTISL. We used the 10Xv2 dataset as an example to further explore the robustness of CTISL concerning different numbers of genes selected by the Chi-square approach. This dataset consists of nine cell types, denoted as $C_{k}$, where $k=1,2,\ldots,9$. For each cell type $C_{k}$, we identified a set of top *t* genes with strong identification ability, denoted as $f_{tk}$. Subsequently, we extracted genes that exclusively appeared in $f_{tk}$ and did not appear in any other $f_{ti}$, where $i\neq k$. The set of genes was denoted as $f_{k}$, representing the distinct genes with stronger identification ability for the cell type $C_{k}$. **Table S4** shows the number of distinct genes for each cell type across various dimensions in the 10Xv2 dataset. Notably, we observed a general reduction in the number of distinct genes for certain cell types with the increase in the number of selected genes. Taking B cells as an example, 369 distinct genes were identified from 1800 selected genes, whereas only 334 distinct genes were identified from 1900 selected genes. Similarly, for CD14+ monocyte, there were 172 distinct genes from 1500 selected genes, but this number decreased to 153 distinct genes when 1600 genes were selected. To visually explore the changing trend of the number of distinct genes specific to each cell type with the increase in the number of selected genes, we further plotted the curve of the growth rate of the number of distinct genes in each cell type along the number of selected genes ranging from 50 to 2000 (**Figure S2**). As shown, when the number of selected genes is above 1000, the number of distinct signature genes for each cell type did not always increase along with the increase of the number of selected genes by the Chi-square approach. A potential reason is that no more additional genes contributed to identifying the corresponding cell type as the number of selected genes increased. Moreover, as the number of selected genes increased, computational times continued to increase, potentially introducing more noise and causing model instability.

**2. Performance evaluation of CTISL on marker gene**

We first extracted marker genes for each cell type from the CellMarker 2.0 database (Hu, et al., 2023). However, we could not find marker genes for cell subtypes in six cross-batch datasets provided in the CellMarker 2.0 database. As a result, we resorted to using the marker genes provided in the original article (Shekhar, et al., 2016; Villani, et al., 2017) for each cell subtype. We then performed the same experiments using marker genes specific to each cell type in seven intra-dataset, seven inter-dataset, six cross-batch, and four cross-species experiments. Results in **Table S6** indicate that CTISL achieved lower performance on marker genes compared to the top 300 genes selected by our approach in terms of average accuracy: 87.8% vs 92.8% in seven intra-dataset experiments, 84.0% vs 87.2% in seven inter-dataset experiments, 92.5% vs 98.3% in six cross-batch experiments, and 78.0% vs 83.5% in four cross-species experiments. Similar trends can be observed in terms of macro F1-score and median F1-score respectively, as shown in **Table S6**. One possible reason for the poorer performance by marker genes is the limited number of known marker genes available for each cell type. For instance, there are only two known marker genes for plasmacytoid dendritic cells (**Table S5**). The limited number of known marker genes poses a challenge in developing a sufficiently reliable model for cell type identification. It should be noted that in the mouse airway dataset, there are three cell types (basal, ciliated, and secretory). While there are five marker genes specific to the basal cell in the CellMarker database, there are no available marker genes for the other two cell types in the CellMarker database. Moreover, five marker genes in the dataset do not appear in the list of overlapping genes between the mouse airway dataset and the human airway dataset. Thus, we did not conduct cross-species prediction from the mouse airway to the human airway.

**3. Performance evaluation by integrating other models into the CTISL framework**

We further explored the performance of CTISL with more base learners, including RF, Gradient Boosting Classifier (Friedman, 2001) (GBC), and Multilayer Perceptron (MLP) (Popescu, et al., 2009). Performance comparison results can be found in **Table S6**.

As aforementioned, CTISL operates as a stacking-based ensemble learning approach. It mainly consists of feature selection (**Figure 1B**) and two layers of ensemble classifiers (**Figure 1C**). In **Figure 1C**, the outputs of the base classifiers in the first layer are used as inputs for the meta-classifier in the second layer. This architecture bears similarities to a two-layer deep learning framework. However, one advantage of the proposed two layers of ensemble classifiers is the flexibility for users to integrate their preferred base learners, allowing the utilization of various heterogeneous classifiers to improve the overall performance. To analyze the ensemble model, we replaced the ensemble classifiers in **Figure 1C** with a two-layer MLP, which employs an output layer with a softmax activation function for predictions. The new model was named Chi-square+MLP. We conducted experiments in different scenarios, including intra-dataset, inter-dataset, cross-batch, and cross-species, using the two-layer MLP. Results in **Table** **S6** demonstrate that the proposed CTISL (i.e., stacking LR and SVM) outperformed Chi-square+MLP in average accuracy (92.8% vs 91.9%) across seven intra-dataset experiments, 87.2% vs 85.4% across seven inter-dataset experiments, 98.3% vs 97.9% across six cross-batch experiments, except across four cross-species experiments (83.5% vs 84.0%). Similar trends can be observed in macro F1-score and median F1-score, as shown in **Table S6**.

Additionally, we conducted the same experiments using MLP with 5000 highly variable genes (HVGs). The results, as presented in **Table S6**, indicate that Chi-square+MLP outperforms MLP with HVGs in average accuracy (91.9% vs 87.2%) across seven intra-dataset experiments, 85.4% vs 85.1% across seven inter-dataset experiments, 97.9% vs 96.6% across six cross-batch experiments, and 84.0% vs 80.3% across four cross-species experiments. Similar comparisons can be observed in the macro F1-score and median F1-score, as shown in **Table S6**.

These results indicate that the ensemble learning model (**Figure 1C**) in CTISL achieved superior performance than Chi-square+MLP, except in cross-species experiments. This denotes that the ensemble learning model in CTISL is effective for identifying cell types. Alternatively, given that Chi-square+MLP achieved better performance than CTISL across cross-species cell type identification, users can select Chi-square+MLP to perform across cross-species cell type identification via our webserver and in the local executable tool. Furthermore, Chi-square+MLP outperformed MLP with HVGs. This denotes that the Chi-square feature selection method is effective for identifying cell types. These results confirm that our structural design of CTISL, including the Chi-square approach for feature gene selection and stacking LR and SVM, is feasible for identifying cell types from single-cell RNA-seq data.

**4. The effect of cell type imbalance on model performance**

In airway datasets, there are three cell types present in both the human airway and mouse airway datasets: Basal, Ciliated, and Secretory. In the human airway dataset, there are 252, 258, and 280 cells of these three cell types, respectively. When trained on the human airway dataset to predict cell types in the mouse airway dataset, all ten predictors achieved better accuracies ranging from 85.1% to 95.1% (**Figure 4**). The mouse airway dataset, however, contains imbalanced sample sizes for the cell types. For the Basal, Ciliated, and Secretory cell types, the numbers of cells are 6009, 1333, and 4792, respectively. When trained on the mouse airway dataset to predict cell types in the human airway dataset, all ten predictors achieved poorer accuracies (**Figure S4**), ranging from 33.0% to 84.7%. However, after down-sampling the three cell types in the mouse dataset (**Section 3.5** in the main text and **Section 3** in the **Supplementary Methods**), there was a significant improvement in accuracies (**Figure S4A**) for six out of the ten predictors, including CTISL, ACTINN, scCapsNet, scmap-cluster, scBERT, and scmap-cell, compared to the original results without down-sampling. This suggests that most predictors, including the proposed CTISL, are sensitive to imbalanced sample sizes of cell types in cross-species cell type prediction.

For the pancreas data, the human dataset consists of 14 cell types, while the mouse dataset contains 13 cell types. Notably, three cell types, acinar, epsilon, and mast, are exclusively present in the human dataset and do not appear in the mouse dataset. Conversely, two cell types, B_cell and immuse_other, exist solely in the mouse dataset and are absent from the human dataset (**Table S7**). Moreover, within the human pancreas dataset, there are 2525 samples of beta cells, which represents the largest sample size, while there are only 7 samples of T cells, indicating the smallest sample size. Similarly, in the mouse pancreas dataset, there are 894 samples of beta cells (maximum sample size) and 6 samples of schwann cells (minimum sample size) (**Table S7**). Consequently, the pancreas dataset exhibits a higher level of cell type imbalance compared to the airway dataset. According to the results in **Figure 4**, when the models were trained on the human pancreas dataset to predict cell types in the mouse pancreas dataset, they achieved poor accuracies ranging from 69.4% to 89.7%, macro F1-scores ranging from 0.347 to 0.536, and median F1-scores ranging from 0.209 to 0.643. Similarly, comparable performances were observed when training on the mouse pancreas dataset to predict cell types in the human pancreas dataset. This means that all ten predictors are sensitive to imbalanced cell numbers in the same cell type across cross-species. The potential reasons include different cell types and gene expression profiles of the same cell type across species (Baron, et al., 2016). Additionally, when conducting the prediction across cross-species on datasets with imbalanced sample sizes, if there are too few samples for the cell type with minimum sample size, the down-sampling strategy may not improve the prediction performance. For example, the human pancreas dataset only has 7 cells of the T cell type while the mouse pancreas dataset has 6 cells of the schwann cells. Consequently, even if we applied the same down-sampling strategy as we did for the mouse airway dataset, except for scmap-cluster in terms of accuracy, all other nine predictors achieved poorer performances compared to those without down-sampling (**Figure S4B-C**).

However, the performance of CTISL and other models seemed robust to other experiment scenarios, despite the imbalanced data. Here we examined the prediction results of two experiment scenarios, including intra-dataset and cross-batch. For the intra-dataset scenario, we examined the 10Xv2 dataset, which exhibits a high degree of sample imbalance according to cell types (**Table S8**). The 10Xv2 dataset consists of nine cell types, with the Cytotoxic+ T cell being the most abundant (2128 cells), and the plasmacytoid dendritic cell as the least abundant (only 38 cells). Interestingly, CTISL achieved high accuracy in predicting the plasmacytoid dendritic cell by correctly classifying 36 out of 38 cells (94.7%), and in predicting the Cytotoxic+ T cell by accurately identifying 1967 out of 2128 cells (92.4%). The overall accuracy of CTISL on the 10Xv2 dataset is 93.2%, outperforming the other nine predictors with accuracies ranging from 78.6% to 92.2% (**Figure 4A**). These results suggest that the prediction performance of CTISL is relatively robust to the imbalanced sample sizes of cell types in intra-dataset experiments. While for the cross-batch scenario, we further examined the Retina(19) dataset (**Table S9**), which comprises 19 cell types and is divided into two batches: Retina(19)_b1 and Retina(19)_b2. Retina(19)_b1 contains 7065 Rod Bipolar cells, but only 9 Cone Photoreceptors, while Retina(19)_b2 contains 3823 Rod Bipolar cells, but 39 Cone Photoreceptors. Surprisingly, all ten predictors achieved accuracies ranging from 87.3% to 97.4% when predicting from Retina(19)_b1 to Retina(19)_b2, with CTISL achieving the second-best accuracy of 96.8% (**Figure 4A**). Furthermore, all ten predictors achieved accuracies ranging from 90.4% to 98.0% when predicting from Retina(19)_b2 to Retina(19)_b1, with CTISL achieving the best accuracy of 98.0% (**Figure 4A**). These results suggest that the prediction performance of CTISL, as well as the other nine predictors, remains relatively robust to the imbalanced sample sizes of cell types in cross-batch experiments.

**5. The scalability of CTISL as the number of cell types increases**

In the default CTISL framework, LR and SVM are trained as base learners for each cell type in the training dataset. By default, CTISL automatically utilizes $t$ LR and $t$ SVM as base learners to train the model, where $t$ represents the number of cell types in the training dataset. Therefore, the number of base learners in CTISL can automatically expand with the increasing number of cell types. As a result, CTISL dynamically increases the number of base learners as the number of cell types increases, which leads to longer training time without compromising the performance of CTISL. To clarify the scalability of CTISL, consider the Retina(19) dataset, which is divided into two batches: Retina(19)_batch1 and Retina(19)_batch2 (**Table S10**). Both batches consist of 19 cell types, including cell subtypes that can be integrated into main cell types. After integrating the cell subtypes into main cell types in Retina(19)_batch1 and Retina(19)_batch2, only six main cell types remain in each dataset (**Table S10**). We refer to these datasets as Retina(6)_batch1 and Retina(6)_batch2, respectively. When training CTISL on Retina(6)_batch1 to predict cell types in Retina(6)_batch2, CTISL automatically trained six LR and SVM models as base learners for each cell type and achieved an accuracy of 97.9%. Similarly, when predicting from Retina(6)_batch2 to Retina(6)_batch1, the accuracy was 98.3%. However, when conducting cross-batch prediction between Retina(19)_batch1 and Retina(19)_batch2, CTISL automatically trained 19 LR and SVM models as base learners for each cell type and achieved a prediction accuracy of 96.8% (**Figure 4A**) when predicting from Retina(19)_batch1 to Retina(19)_batch2, and 98.0% (**Figure 4A**) when predicting from Retina(19)_batch2 to Retina(19)_batch1. Therefore, CTISL can automatically adjust the number of base learners based on the number of cell types in the training dataset. However, it is important to note that more cell types in the training dataset result in longer computational times.

To enable users to explore the prediction performance using different base learners, we have developed a user-friendly webserver at http://bigdata.biocie.cn/CTISLweb/home. This server enables users to use the trained models to predict cell types using their datasets and to choose different base learners. Through this webserver, users can conveniently select their preferred base learners for training models on both public and individual datasets.

**Supplementary Table S1.** scRNA-seq datasets collected for training and evaluating CTISL and state-of-the-art methods

| Species | Platform | Dataset | #Cells and #genes | #Cell types | Experiment scenario |
| --- | --- | --- | --- | --- | --- |
| *H. sapiens* | 10Xv2 | 10Xv2 | 6444, 22316 | 9 | Intra-dataset  Inter-dataset |
|  | 10Xv3 | 10Xv3 | 3222, 21939 | 8 | Intra-dataset  Inter-dataset |
|  | CEL-Seq2 | CELSeq | 253, 20066 | 7 | Intra-dataset  Inter-dataset |
|  | Drop-Seq | DropSeq | 3222, 19954 | 9 | Intra-dataset  Inter-dataset |
|  | inDrop | inDrop | 3222, 17188 | 7 | Intra-dataset  Inter-dataset |
|  | SMARTSeq2 | SMARTSeq2 | 253, 22654 | 6 | Intra-dataset  Inter-dataset |
|  | SeqWell | SeqWell | 3176, 21200 | 7 | Intra-dataset  Inter-dataset |
|  | inDrop | HumanPancreas | 8569, 16381 | 14 | Cross-species |
|  | inDrop | HumanAirway | 790, 15733 | 3 | Cross-species |
|  | SMART-Seq2 | Dendritic_batch1 | 384, 15426 | 4 | Cross-batch |
|  | SMART-Seq2 | Dendritic_batch2 | 384, 15426 | 4 | Cross-batch |
| *M*. *musculus* | inDrop | MousePancreas | 1886, 14878 | 13 | Cross-species |
|  | inDrop | MouseAirway | 12134, 15733 | 3 | Cross-species |
|  | Drop-Seq | Retina(5)_batch1 | 13666, 13166 | 5 | Cross-batch |
|  | Drop-Seq | Retina(5)_batch2 | 13164, 13166 | 5 | Cross-batch |
|  | Drop-Seq | Retina(19)_batch1 | 13987, 13166 | 19 | Cross-batch |
|  | Drop-Seq | Retina(19)_batch2 | 13512, 13166 | 19 | Cross-batch |

**Supplementary Table S2**. Performance comparison among different feature selection methods using SVM

| Score | Method | 10Xv2 | 10Xv3 | CELSeq | DropSeq | inDrop | SMARTSeq2 | SeqWell | Average |
| --- | --- | --- | --- | --- | --- | --- | --- | --- | --- |
| Accuracy | Chi-square (100) | 0.930 | 0.944 | 0.901 | 0.902 | 0.899 | 0.933 | 0.867 | **0.911** |
|  | Chi-square (200) | 0.931 | 0.945 | 0.913 | 0.893 | 0.895 | 0.921 | 0.871 | 0.910 |
|  | Chi-square (300) | 0.928 | 0.943 | 0.905 | 0.888 | 0.892 | 0.921 | 0.869 | 0.907 |
|  | Chi-square (400) | 0.928 | 0.943 | 0.893 | 0.883 | 0.890 | 0.897 | 0.867 | 0.900 |
|  | Chi-square (500) | 0.927 | 0.941 | 0.889 | 0.880 | 0.890 | 0.889 | 0.868 | 0.898 |
|  | HVG (2000) | 0.848 | 0.906 | 0.641 | 0.815 | 0.773 | 0.640 | 0.787 | 0.773 |
|  | HVG (3000) | 0.857 | 0.900 | 0.648 | 0.831 | 0.802 | 0.644 | 0.792 | 0.782 |
|  | HVG (4000) | 0.862 | 0.902 | 0.640 | 0.843 | 0.800 | 0.648 | 0.800 | 0.785 |
|  | HVG (5000) | 0.859 | 0.895 | 0.605 | 0.842 | 0.808 | 0.648 | 0.806 | 0.780 |
|  | Limma (100) | 0.933 | 0.944 | 0.905 | 0.899 | 0.902 | 0.937 | 0.827 | 0.907 |
|  | Limma (200) | 0.931 | 0.944 | 0.901 | 0.893 | 0.893 | 0.929 | 0.834 | 0.904 |
|  | Limma (300) | 0.930 | 0.946 | 0.889 | 0.889 | 0.892 | 0.921 | 0.830 | 0.900 |
|  | Limma (400) | 0.928 | 0.942 | 0.869 | 0.888 | 0.890 | 0.913 | 0.830 | 0.894 |
|  | Limma (500) | 0.927 | 0.942 | 0.842 | 0.887 | 0.885 | 0.901 | 0.828 | 0.887 |
|  | GeneClust | 0.882 | 0.898 | 0.716 | 0.782 | 0.770 | 0.909 | 0.760 | 0.817 |
| Macro F1-score | Chi-square (100) | 0.936 | 0.946 | 0.792 | 0.765 | 0.692 | 0.860 | 0.609 | 0.800 |
|  | Chi-square (200) | 0.927 | 0.943 | 0.808 | 0.728 | 0.656 | 0.788 | 0.608 | 0.780 |
|  | Chi-square (300) | 0.921 | 0.933 | 0.802 | 0.718 | 0.654 | 0.788 | 0.597 | 0.773 |
|  | Chi-square (400) | 0.920 | 0.933 | 0.796 | 0.711 | 0.653 | 0.773 | 0.595 | 0.769 |
|  | Chi-square (500) | 0.910 | 0.923 | 0.794 | 0.705 | 0.653 | 0.769 | 0.590 | 0.763 |
|  | HVG (2000) | 0.660 | 0.781 | 0.544 | 0.589 | 0.547 | 0.430 | 0.488 | 0.577 |
|  | HVG (3000) | 0.636 | 0.759 | 0.556 | 0.590 | 0.571 | 0.438 | 0.478 | 0.575 |
|  | HVG (4000) | 0.631 | 0.751 | 0.518 | 0.588 | 0.569 | 0.446 | 0.489 | 0.570 |
|  | HVG (5000) | 0.617 | 0.734 | 0.421 | 0.586 | 0.577 | 0.446 | 0.484 | 0.552 |
|  | Limma (100) | 0.942 | 0.939 | 0.785 | 0.819 | 0.726 | 0.863 | 0.536 | **0.801** |
|  | Limma (200) | 0.935 | 0.939 | 0.788 | 0.797 | 0.662 | 0.792 | 0.541 | 0.779 |
|  | Limma (300) | 0.929 | 0.938 | 0.771 | 0.776 | 0.657 | 0.787 | 0.520 | 0.768 |
|  | Limma (400) | 0.926 | 0.932 | 0.739 | 0.762 | 0.653 | 0.783 | 0.519 | 0.759 |
|  | Limma (500) | 0.921 | 0.933 | 0.668 | 0.743 | 0.649 | 0.776 | 0.511 | 0.743 |
|  | GeneClust | 0.814 | 0.841 | 0.565 | 0.635 | 0.538 | 0.811 | 0.479 | 0.669 |
| Median F1-score | Chi-square (100) | 0.961 | 0.982 | 0.937 | 0.864 | 0.917 | 0.956 | 0.841 | **0.923** |
|  | Chi-square (200) | 0.952 | 0.979 | 0.956 | 0.866 | 0.912 | 0.931 | 0.823 | 0.917 |
|  | Chi-square (300) | 0.954 | 0.971 | 0.944 | 0.871 | 0.911 | 0.931 | 0.771 | 0.908 |
|  | Chi-square (400) | 0.952 | 0.970 | 0.933 | 0.873 | 0.911 | 0.931 | 0.768 | 0.905 |
|  | Chi-square (500) | 0.944 | 0.962 | 0.930 | 0.871 | 0.912 | 0.902 | 0.768 | 0.898 |
|  | HVG (2000) | 0.848 | 0.927 | 0.669 | 0.820 | 0.802 | 0.381 | 0.770 | 0.745 |
|  | HVG (3000) | 0.864 | 0.913 | 0.727 | 0.812 | 0.832 | 0.366 | 0.705 | 0.746 |
|  | HVG (4000) | 0.869 | 0.910 | 0.662 | 0.830 | 0.829 | 0.366 | 0.739 | 0.744 |
|  | HVG (5000) | 0.868 | 0.899 | 0.420 | 0.842 | 0.836 | 0.366 | 0.683 | 0.702 |
|  | Limma (100) | 0.978 | 0.976 | 0.937 | 0.895 | 0.914 | 0.968 | 0.664 | 0.905 |
|  | Limma (200) | 0.959 | 0.978 | 0.928 | 0.886 | 0.913 | 0.942 | 0.672 | 0.897 |
|  | Limma (300) | 0.947 | 0.980 | 0.899 | 0.885 | 0.912 | 0.928 | 0.602 | 0.879 |
|  | Limma (400) | 0.949 | 0.978 | 0.871 | 0.887 | 0.909 | 0.917 | 0.600 | 0.873 |
|  | Limma (500) | 0.946 | 0.979 | 0.858 | 0.877 | 0.905 | 0.908 | 0.576 | 0.864 |
|  | GeneClust | 0.880 | 0.908 | 0.660 | 0.770 | 0.700 | 0.932 | 0.591 | 0.777 |

Note: the number in the bracket in the second column denotes the number of genes selected by the corresponding method.

**Supplementary Table S3**. Performance comparison among different feature selection methods using LR

| Score | Method | 10Xv2 | 10Xv3 | CELSeq | DropSeq | inDrop | SMARTSeq2 | SeqWell | Average |
| --- | --- | --- | --- | --- | --- | --- | --- | --- | --- |
| Accuracy | Chi-square (100) | 0.912 | 0.940 | 0.917 | 0.907 | 0.887 | 0.952 | 0.846 | 0.909 |
|  | Chi-square (200) | 0.911 | 0.941 | 0.913 | 0.920 | 0.905 | 0.952 | 0.872 | 0.916 |
|  | Chi-square (300) | 0.920 | 0.945 | 0.909 | 0.919 | 0.904 | 0.952 | 0.865 | 0.916 |
|  | Chi-square (400) | 0.920 | 0.945 | 0.909 | 0.914 | 0.908 | 0.960 | 0.873 | **0.918** |
|  | Chi-square (500) | 0.920 | 0.947 | 0.905 | 0.914 | 0.904 | 0.960 | 0.877 | **0.918** |
|  | HVG (2000) | 0.878 | 0.935 | 0.818 | 0.843 | 0.817 | 0.877 | 0.819 | 0.855 |
|  | HVG (3000) | 0.894 | 0.943 | 0.842 | 0.868 | 0.857 | 0.881 | 0.838 | 0.875 |
|  | HVG (4000) | 0.906 | 0.948 | 0.873 | 0.885 | 0.876 | 0.897 | 0.855 | 0.891 |
|  | HVG (5000) | 0.910 | 0.947 | 0.877 | 0.886 | 0.885 | 0.901 | 0.854 | 0.894 |
|  | Limma (100) | 0.910 | 0.937 | 0.905 | 0.901 | 0.893 | 0.960 | 0.792 | 0.900 |
|  | Limma (200) | 0.917 | 0.941 | 0.917 | 0.908 | 0.897 | 0.952 | 0.826 | 0.908 |
|  | Limma (300) | 0.919 | 0.947 | 0.933 | 0.912 | 0.898 | 0.948 | 0.831 | 0.913 |
|  | Limma (400) | 0.919 | 0.949 | 0.913 | 0.914 | 0.901 | 0.941 | 0.839 | 0.911 |
|  | Limma (500) | 0.920 | 0.949 | 0.909 | 0.913 | 0.897 | 0.937 | 0.847 | 0.910 |
|  | GeneClust | 0.887 | 0.914 | 0.806 | 0.788 | 0.769 | 0.944 | 0.753 | 0.837 |
| Macro F1-score | Chi-square (100) | 0.936 | 0.950 | 0.871 | 0.891 | 0.756 | 0.960 | 0.829 | 0.885 |
|  | Chi-square (200) | 0.933 | 0.947 | 0.854 | 0.899 | 0.796 | 0.959 | 0.831 | **0.888** |
|  | Chi-square (300) | 0.937 | 0.951 | 0.823 | 0.901 | 0.754 | 0.960 | 0.814 | 0.877 |
|  | Chi-square (400) | 0.936 | 0.948 | 0.823 | 0.893 | 0.757 | 0.964 | 0.817 | 0.877 |
|  | Chi-square (500) | 0.933 | 0.949 | 0.821 | 0.894 | 0.753 | 0.964 | 0.807 | 0.874 |
|  | HVG (2000) | 0.899 | 0.942 | 0.730 | 0.827 | 0.647 | 0.758 | 0.742 | 0.792 |
|  | HVG (3000) | 0.907 | 0.943 | 0.763 | 0.835 | 0.679 | 0.759 | 0.755 | 0.806 |
|  | HVG (4000) | 0.915 | 0.948 | 0.791 | 0.850 | 0.701 | 0.808 | 0.778 | 0.827 |
|  | HVG (5000) | 0.922 | 0.946 | 0.792 | 0.851 | 0.716 | 0.809 | 0.773 | 0.830 |
|  | Limma (100) | 0.930 | 0.940 | 0.809 | 0.887 | 0.820 | 0.964 | 0.711 | 0.866 |
|  | Limma (200) | 0.935 | 0.945 | 0.826 | 0.894 | 0.785 | 0.960 | 0.753 | 0.871 |
|  | Limma (300) | 0.934 | 0.950 | 0.846 | 0.897 | 0.752 | 0.958 | 0.770 | 0.872 |
|  | Limma (400) | 0.934 | 0.949 | 0.822 | 0.896 | 0.758 | 0.941 | 0.759 | 0.866 |
|  | Limma (500) | 0.934 | 0.948 | 0.821 | 0.895 | 0.752 | 0.939 | 0.762 | 0.864 |
|  | GeneClust | 0.898 | 0.922 | 0.724 | 0.764 | 0.610 | 0.955 | 0.613 | 0.784 |
| Median F1-score | Chi-square (100) | 0.970 | 0.985 | 0.945 | 0.910 | 0.885 | 0.989 | 0.872 | 0.937 |
|  | Chi-square (200) | 0.970 | 0.981 | 0.946 | 0.916 | 0.903 | 0.983 | 0.877 | **0.939** |
|  | Chi-square (300) | 0.966 | 0.981 | 0.946 | 0.932 | 0.905 | 0.989 | 0.846 | 0.938 |
|  | Chi-square (400) | 0.969 | 0.980 | 0.946 | 0.922 | 0.912 | 0.991 | 0.851 | **0.939** |
|  | Chi-square (500) | 0.965 | 0.981 | 0.937 | 0.924 | 0.908 | 0.991 | 0.848 | 0.936 |
|  | HVG (2000) | 0.941 | 0.977 | 0.824 | 0.861 | 0.813 | 0.898 | 0.800 | 0.873 |
|  | HVG (3000) | 0.946 | 0.972 | 0.910 | 0.881 | 0.861 | 0.903 | 0.822 | 0.899 |
|  | HVG (4000) | 0.955 | 0.983 | 0.939 | 0.902 | 0.883 | 0.937 | 0.842 | 0.920 |
|  | HVG (5000) | 0.964 | 0.984 | 0.914 | 0.907 | 0.890 | 0.944 | 0.810 | 0.916 |
|  | Limma (100) | 0.965 | 0.966 | 0.946 | 0.891 | 0.898 | 0.991 | 0.780 | 0.920 |
|  | Limma (200) | 0.971 | 0.979 | 0.963 | 0.907 | 0.899 | 0.982 | 0.791 | 0.927 |
|  | Limma (300) | 0.969 | 0.986 | 0.985 | 0.917 | 0.900 | 0.982 | 0.770 | 0.930 |
|  | Limma (400) | 0.966 | 0.985 | 0.961 | 0.921 | 0.900 | 0.962 | 0.777 | 0.925 |
|  | Limma (500) | 0.966 | 0.986 | 0.952 | 0.909 | 0.898 | 0.962 | 0.798 | 0.924 |
|  | GeneClust | 0.937 | 0.963 | 0.810 | 0.778 | 0.757 | 0.982 | 0.641 | 0.838 |

Note: the number in the bracket in the second column denotes the number of genes selected by the corresponding method.

**Supplementary Table S4.** The number of distinct genes for each cell type in the 10Xv2 dataset by the Chi-square approach

| Number of selected genes | B cell | CD14+  monocyte | CD16+  monocyte | CD4+  T cell | Cytotoxic T cell | Dendritic cell | megakaryocyte | Natural killer cell | Plasmacytoid dendritic cell |
| --- | --- | --- | --- | --- | --- | --- | --- | --- | --- |
| 10 | 10 | 9 | 10 | 10 | 8 | 10 | 10 | 9 | 10 |
| 50 | 41 | 21 | 39 | 27 | 12 | 35 | 49 | 36 | 45 |
| 100 | 69 | 31 | 63 | 61 | 19 | 59 | 98 | 65 | 81 |
| 200 | 100 | 66 | 107 | 96 | 30 | 98 | 190 | 112 | 140 |
| 300 | 136 | 78 | 155 | 124 | 37 | 133 | 258 | 164 | 197 |
| 400 | 155 | 85 | 203 | 142 | 36 | 170 | 301 | 197 | 257 |
| 500 | 181 | 89 | 230 | 164 | 44 | 200 | 326 | 248 | 308 |
| 600 | 203 | 110 | 246 | 184 | 64 | 233 | 348 | 285 | 358 |
| 700 | 226 | 118 | 258 | 206 | 61 | 252 | 353 | 310 | 408 |
| 800 | 243 | 121 | 279 | 215 | 67 | 268 | 358 | 339 | 449 |
| 900 | 262 | 129 | 305 | 230 | 72 | 304 | 352 | 362 | 487 |
| 1000 | 284 | 132 | 323 | 236 | 86 | 315 | 351 | 390 | 532 |
| 1100 | 289 | 140 | 343 | 252 | 99 | 309 | 349 | 430 | 570 |
| 1200 | 297 | 142 | 367 | 270 | 110 | 314 | 341 | 451 | 579 |
| 1300 | 308 | 154 | 378 | 294 | 121 | 326 | 330 | 473 | 628 |
| 1400 | 302 | 158 | 404 | 297 | 130 | 340 | 312 | 503 | 621 |
| 1500 | 324 | 172 | 397 | 313 | 132 | 352 | 305 | 512 | 691 |
| 1600 | 325 | 153 | 410 | 332 | 132 | 374 | 291 | 525 | 694 |
| 1700 | 322 | 171 | 435 | 333 | 152 | 359 | 280 | 539 | 720 |
| 1800 | 369 | 170 | 420 | 340 | 140 | 365 | 277 | 542 | 692 |
| 1900 | 334 | 164 | 437 | 350 | 149 | 362 | 270 | 577 | 726 |
| 2000 | 349 | 152 | 471 | 380 | 165 | 366 | 265 | 596 | 731 |

**Supplementary Table S5.** A comparison between the selected feature genes by the Chi-square method and the marker genes extracted from the CellMarker 2.0 database (Hu, et al., 2023) for each cell type in the PBMC-10Xv2 dataset

| **Cell type** | **Marker gene** |
| --- | --- |
| B cell | AHNAK, AIM2, **BACH2**, **BCL11A**, **BLNK**, CALR, CD11C, CD127, **CD19**, CD1C, CD20, CD21, **CD22**, **CD24**, CD25, CD27, **CD37**, CD38, **CD40**, CD45, CD5, CD72, **CD74**, CD79, **CD79A, CD79B**, CD81, CD95, CR2, **FCER2**, FCGR2A, **FCRL1**, **FCRLA**, HLA-A, **HLA-DPB1**, IGD, **IGHA1**, **IGHD**, IGHG2, **IGHM**, IGJ, **IGKC**, IGM, **IL4R**, **ITGB1**, **JCHAIN**, LEU-12, LILRA4, **MS4A1**, **MZB1**, NFKBIA, **PAX5**, **PLPP5**, **SPIB**, **SWAP70**, T-BET, **TCL1A**, **TNFRSF13C**, TNFRSF17, XBP1, **YBX3**, ZAP70 |
| CD14+ monocyte | **CD14**, **CD4**, FCGR3A, **LYZ**, NR4A1, **S100A9**, SL00A8, SL00A9 |
| CD16+ monocyte | CD14, CD16, CD4, CX3CR1, **FCGR3A**, **LYZ**, **MS4A7**, NR4A1 |
| CD4+T cell | ACTB, ACTG1, **CCR7**, CD127, CD154, **CD27**, **CD28**, CD3, **CD3D**, **CD3E**, CD3G, CD4, **CD44**, CD45, CTLA4, FOXP3, GLUT1, IL12RB1, IL2RA, IL4R, **IL7R**, **LTB**, MYO1G, PFN1, **RORA**, S100A10, **S100A11**, S100A4, SELL, STAT4 |
| Cytotoxic T cell | CD11B, **CD3D**, CD8, **CD8A**, **CD8B**, **GNLY**, **GZMA**, **GZMB**, **KLRG1**, LEU-2A, **PRF1** |
| Dendritic cell | BMP4, CD11C, CD141, **CD1C**, **CD1E**, CD80, CD83, **CD86**, **CLEC10A**, CLEC12A, CLEC4C, **CST3**, CYBA, **FCER1A**, HSPA5, IFI6, **IFITM3**, IGJ, IL3RA, IRF7, **IRF8**, ISG15, LILRA4, **LILRB4**, MHC CLASS II, MZB1, NRP1, **PLD4**, SOX2, UBC |
| Megakaryocyte | CD34, CD41A, CD42A, CD61, CD79A, **GP9**, **ITGA2B**, **MYL9**, **PF4**, **PPBP**, **TUBB1** |
| Natural killer cell | **CCL3**, **CCL4**, **CCL5**, CD16, **CD160**, CD161, **CD247**, CD25, CD3, CD337, **CD38**, **CD3D**, CD3E, **CD3G**, CD45, CD49A, CD49E, CD52, CD56, CD57, CD62L, CD69, **CD7**, CD94, **CHST2**, CX3CR1, CXCR6, CXXC5, **FCER1G**, **FCGR3A**, FCGR3B, FOS, **GNLY**, **GZMB**, IL18RAP, **IL2RB**, **KIR2DL1**, KIR2DS1, **KLRB1**, **KLRC1**, **KLRD1**, **KLRF1**, KLRK1, NCAM, **NCAM1**, NKG2A, NKG2C, NKG2D, **NKG7**, NKP46, NKP80, **PRSS23**, SELL, **SPON2**, **TRAC**, **TRDC**, **TRGC1**, TRGC2, **TYROBP**, **ZBTB16**, ZFP36 |
| Plasmacytoid dendritic cell | CD123, **GZMB** |

Note: bolded genes denote that these marker genes are contained in the selected gene list (top 300 genes) by the Chi-square method.

**Supplementary Table S6.** Performance evaluation of various models, including CTISL, LR, SVM, CTISL with marker genes, Chi-square+MLP, MLP+HVG(5000), and CTISL with LR+SVM+RF+GBC

| Score | Evaluation Strategies | Dataset | CTISL | LR | SVM | CTISL With  marker genes | Chi-square  +MLP | MLP+HVG  (5000) | CTISLwith LR+  SVM+RF+GBC |
| --- | --- | --- | --- | --- | --- | --- | --- | --- | --- |
| Accuracy | Intra-dataset | 10Xv2 | 0.932 | 0.920 | 0.928 | 0.916 | 0.923 | 0.903 | 0.932 |
|  | Intra-dataset | 10Xv3 | 0.951 | 0.945 | 0.943 | 0.938 | 0.949 | 0.944 | 0.949 |
|  | Intra-dataset | CELSeq | 0.929 | 0.909 | 0.905 | 0.822 | 0.905 | 0.795 | 0.925 |
|  | Intra-dataset | DropSeq | 0.931 | 0.919 | 0.888 | 0.885 | 0.926 | 0.878 | 0.931 |
|  | Intra-dataset | inDrop | 0.916 | 0.904 | 0.892 | 0.836 | 0.903 | 0.863 | 0.917 |
|  | Intra-dataset | SMARTSeq2 | 0.964 | 0.952 | 0.921 | 0.933 | 0.956 | 0.869 | 0.968 |
|  | Intra-dataset | SeqWell | 0.875 | 0.865 | 0.869 | 0.817 | 0.874 | 0.855 | 0.873 |
|  | **Intra-dataset** | **Average** | **0.928** | 0.916 | 0.907 | 0.878 | 0.919 | 0.872 | **0.928** |
|  | Inter-dataset | 10Xv2 (as the testing data) | 0.901 | 0.872 | 0.887 | 0.906 | 0.902 | 0.866 | 0.895 |
|  | Inter-dataset | 10Xv3 (as the testing data) | 0.924 | 0.903 | 0.915 | 0.926 | 0.922 | 0.904 | 0.918 |
|  | Inter-dataset | CELSeq (as the testing data) | 0.889 | 0.798 | 0.866 | 0.850 | 0.842 | 0.862 | 0.881 |
|  | Inter-dataset | DropSeq (as the testing data) | 0.870 | 0.805 | 0.863 | 0.801 | 0.840 | 0.845 | 0.860 |
|  | Inter-dataset | inDrop (as the testing data) | 0.856 | 0.777 | 0.877 | 0.792 | 0.825 | 0.819 | 0.838 |
|  | Inter-dataset | SMARTSeq2 (as the testing data) | 0.921 | 0.909 | 0.921 | 0.858 | 0.901 | 0.909 | 0.917 |
|  | Inter-dataset | SeqWell (as the testing data) | 0.741 | 0.745 | 0.77 | 0.748 | 0.744 | 0.752 | 0.749 |
|  | **Inter-dataset** | **Average** | **0.872** | 0.830 | 0.871 | 0.840 | 0.854 | 0.851 | 0.865 |
|  | Cross-batch | Retina (5) batch (1 to 2) | 0.996 | 0.991 | 0.975 | 0.992 | 0.993 | 0.994 | 0.994 |
|  | Cross-batch | Retina (5) batch (2 to 1) | 1 | 1 | 0.997 | 0.999 | 0.999 | 0.999 | 1 |
|  | Cross-batch | Retina (19) batch (1 to 2) | 0.968 | 0.958 | 0.917 | 0.777 | 0.958 | 0.905 | 0.965 |
|  | Cross-batch | Retina (19) batch (2 to 1) | 0.980 | 0.981 | 0.982 | 0.852 | 0.981 | 0.975 | 0.957 |
|  | Cross-batch | Dendritic batch (1 to 2) | 0.971 | 0.964 | 0.956 | 0.969 | 0.966 | 0.953 | 0.964 |
|  | Cross-batch | Dendritic batch (2 to 1) | 0.981 | 0.976 | 0.971 | 0.958 | 0.979 | 0.968 | 0.981 |
|  | **Cross-batch** | **Average** | **0.983** | 0.978 | 0.966 | 0.925 | 0.979 | 0.966 | 0.977 |
|  | Cross-species | Pancreas (human to mouse) | 0.843 | 0.858 | 0.867 | 0.745 | 0.828 | 0.786 | 0.863 |
|  | Cross-species | Pancreas (mouse to human) | 0.577 | 0.538 | 0.541 | 0.757 | 0.606 | 0.576 | 0.541 |
|  | Cross-species | Airway (human to mouse) | 0.924 | 0.949 | 0.929 | 0.838 | 0.934 | 0.862 | 0.922 |
|  | Cross-species | Airway (mouse to human) | 0.997 | 0.996 | 0.997 | - | 0.993 | 0.989 | 0.997 |
|  | **Cross-species** | **Average** | 0.835 | 0.835 | 0.834 | 0.780 | **0.840** | 0.803 | 0.831 |
| Macro F1-score | Intra-dataset | 10Xv2 | 0.943 | 0.937 | 0.921 | 0.930 | 0.936 | 0.910 | 0.946 |
|  | Intra-dataset | 10Xv3 | 0.949 | 0.951 | 0.933 | 0.933 | 0.957 | 0.944 | 0.953 |
|  | Intra-dataset | CELSeq | 0.911 | 0.823 | 0.802 | 0.735 | 0.860 | 0.697 | 0.891 |
|  | Intra-dataset | DropSeq | 0.928 | 0.901 | 0.718 | 0.848 | 0.903 | 0.833 | 0.925 |
|  | Intra-dataset | inDrop | 0.802 | 0.754 | 0.654 | 0.733 | 0.772 | 0.664 | 0.815 |
|  | Intra-dataset | SMARTSeq2 | 0.978 | 0.960 | 0.788 | 0.959 | 0.974 | 0.781 | 0.981 |
|  | Intra-dataset | SeqWell | 0.834 | 0.814 | 0.597 | 0.725 | 0.825 | 0.753 | 0.838 |
|  | **Intra-dataset** | **Average** | 0.906 | 0.877 | 0.773 | 0.838 | 0.890 | 0.797 | **0.907** |
|  | Inter-dataset | 10Xv2 (as the testing data) | 0.877 | 0.852 | 0.825 | 0.858 | 0.863 | 0.861 | 0.865 |
|  | Inter-dataset | 10Xv3 (as the testing data) | 0.818 | 0.789 | 0.803 | 0.807 | 0.816 | 0.791 | 0.809 |
|  | Inter-dataset | CELSeq (as the testing data) | 0.756 | 0.651 | 0.719 | 0.726 | 0.710 | 0.740 | 0.741 |
|  | Inter-dataset | DropSeq (as the testing data) | 0.783 | 0.740 | 0.713 | 0.633 | 0.775 | 0.767 | 0.769 |
|  | Inter-dataset | inDrop (as the testing data) | 0.630 | 0.587 | 0.669 | 0.586 | 0.636 | 0.611 | 0.630 |
|  | Inter-dataset | SMARTSeq2 (as the testing data) | 0.647 | 0.642 | 0.647 | 0.634 | 0.643 | 0.643 | 0.646 |
|  | Inter-dataset | SeqWell (as the testing data) | 0.624 | 0.609 | 0.633 | 0.521 | 0.619 | 0.624 | 0.617 |
|  | **Inter-dataset** | **Average** | **0.734** | 0.696 | 0.716 | 0.681 | 0.723 | 0.720 | 0.725 |
|  | Cross-batch | Retina (5) batch (1 to 2) | 0.931 | 0.879 | 0.386 | 0.921 | 0.913 | 0.914 | 0.905 |
|  | Cross-batch | Retina (5) batch (2 to 1) | 0.982 | 0.977 | 0.732 | 0.971 | 0.976 | 0.984 | 0.994 |
|  | Cross-batch | Retina (19) batch (1 to 2) | 0.951 | 0.935 | 0.79 | 0.673 | 0.931 | 0.841 | 0.947 |
|  | Cross-batch | Retina (19) batch (2 to 1) | 0.951 | 0.956 | 0.953 | 0.692 | 0.954 | 0.935 | 0.930 |
|  | Cross-batch | Dendritic batch (1 to 2) | 0.971 | 0.963 | 0.956 | 0.969 | 0.966 | 0.953 | 0.963 |
|  | Cross-batch | Dendritic batch (2 to 1) | 0.981 | 0.976 | 0.971 | 0.958 | 0.979 | 0.968 | 0.981 |
|  | **Cross-batch** | **Average** | **0.961** | 0.948 | 0.798 | 0.864 | 0.953 | 0.933 | 0.953 |
|  | Cross-species | Pancreas (human to mouse) | 0.432 | 0.483 | 0.463 | 0.430 | 0.545 | 0.450 | 0.538 |
|  | Cross-species | Pancreas (mouse to human) | 0.358 | 0.354 | 0.329 | 0.441 | 0.451 | 0.385 | 0.386 |
|  | Cross-species | Airway (human to mouse) | 0.928 | 0.948 | 0.925 | 0.867 | 0.933 | 0.879 | 0.922 |
|  | Cross-species | Airway (mouse to human) | 0.998 | 0.996 | 0.997 | - | 0.992 | 0.991 | 0.997 |
|  | **Cross-species** | **Average** | 0.679 | 0.695 | 0.678 | 0.579 | **0.730** | 0.676 | 0.711 |
| Median F1-score | Intra-dataset | 10Xv2 | 0.961 | 0.966 | 0.954 | 0.951 | 0.961 | 0.938 | 0.946 |
|  | Intra-dataset | 10Xv3 | 0.986 | 0.981 | 0.971 | 0.961 | 0.986 | 0.965 | 0.983 |
|  | Intra-dataset | CELSeq | 0.955 | 0.946 | 0.944 | 0.842 | 0.935 | 0.747 | 0.965 |
|  | Intra-dataset | DropSeq | 0.948 | 0.932 | 0.871 | 0.873 | 0.928 | 0.904 | 0.938 |
|  | Intra-dataset | inDrop | 0.913 | 0.905 | 0.911 | 0.830 | 0.909 | 0.878 | 0.919 |
|  | Intra-dataset | SMARTSeq2 | 0.989 | 0.989 | 0.931 | 0.983 | 0.988 | 0.896 | 0.992 |
|  | Intra-dataset | SeqWell | 0.874 | 0.846 | 0.771 | 0.753 | 0.848 | 0.828 | 0.877 |
|  | **Intra-dataset** | **Average** | **0.947** | 0.938 | 0.908 | 0.885 | 0.936 | 0.879 | 0.946 |
|  | Inter-dataset | 10Xv2 (as the testing data) | 0.901 | 0.873 | 0.94 | 0.891 | 0.887 | 0.913 | 0.886 |
|  | Inter-dataset | 10Xv3 (as the testing data) | 0.912 | 0.893 | 0.915 | 0.919 | 0.916 | 0.910 | 0.907 |
|  | Inter-dataset | CELSeq (as the testing data) | 0.859 | 0.769 | 0.805 | 0.840 | 0.827 | 0.842 | 0.845 |
|  | Inter-dataset | DropSeq (as the testing data) | 0.885 | 0.800 | 0.756 | 0.613 | 0.840 | 0.854 | 0.871 |
|  | Inter-dataset | inDrop (as the testing data) | 0.752 | 0.627 | 0.818 | 0.714 | 0.727 | 0.681 | 0.705 |
|  | Inter-dataset | SMARTSeq2 (as the testing data) | 0.931 | 0.906 | 0.931 | 0.893 | 0.922 | 0.911 | 0.924 |
|  | Inter-dataset | SeqWell (as the testing data) | 0.762 | 0.738 | 0.783 | 0.568 | 0.766 | 0.740 | 0.718 |
|  | **Inter-dataset** | **Average** | **0.857** | 0.801 | 0.850 | 0.777 | 0.841 | 0.836 | 0.837 |
|  | Cross-batch | Retina (5) batch (1 to 2) | 0.952 | 0.976 | 0.000 | 0.969 | 0.961 | 0.984 | 0.930 |
|  | Cross-batch | Retina (5) batch (2 to 1) | 0.989 | 0.977 | 0.84 | 0.996 | 0.978 | 0.997 | 1 |
|  | Cross-batch | Retina (19) batch (1 to 2) | 0.968 | 0.959 | 0.927 | 0.735 | 0.955 | 0.911 | 0.963 |
|  | Cross-batch | Retina (19) batch (2 to 1) | 0.974 | 0.978 | 0.974 | 0.731 | 0.971 | 0.966 | 0.961 |
|  | Cross-batch | Dendritic batch (1 to 2) | 0.974 | 0.962 | 0.954 | 0.974 | 0.964 | 0.952 | 0.964 |
|  | Cross-batch | Dendritic batch (2 to 1) | 0.982 | 0.973 | 0.97 | 0.959 | 0.979 | 0.969 | 0.982 |
|  | **Cross-batch** | **Average** | **0.973** | 0.971 | 0.778 | 0.894 | 0.968 | 0.963 | 0.967 |
|  | Cross-species | Pancreas (human to mouse) | 0.494 | 0.619 | 0.642 | 0.429 | 0.609 | 0.535 | 0.616 |
|  | Cross-species | Pancreas (mouse to human) | 0.174 | 0.098 | 0.108 | 0.460 | 0.520 | 0.304 | 0.280 |
|  | Cross-species | Airway (human to mouse) | 0.933 | 0.948 | 0.917 | 0.825 | 0.935 | 0.860 | 0.924 |
|  | Cross-species | Airway (mouse to human) | 0.998 | 0.996 | 0.995 | - | 0.989 | 0.987 | 0.996 |
|  | **Cross-species** | **Average** | 0.650 | 0.665 | 0.666 | 0.571 | **0.763** | 0.672 | 0.704 |

Note: The column labeled ‘Chi-square+MLP’ denotes that the ensemble model (**Figure 1C**) in CTISL is replaced with MLP. Similarly, the column labeled ‘CTISL with LR+SVM+ RF+GBC’ denotes that the ensemble model (**Figure 1C**) in CTISL consists of LR, SVM, RF, and GradientBoostingClassifier (GBC). The column labeled ‘CTISL with marker genes’ denotes that the ensemble learning model in CTISL uses marker genes as input. The column labeled ‘MLP+HVG(5000)’ denotes the MLP model using the 5000 highly variable genes as input. Refer to **Section 1** of **Supplementary Methods** for more details of these CTISL variations. Bolded numbers denote the best performance in the corresponding rows. For the mouse airway dataset, we performed downsampling to balance the class distribution in the dataset (**Section 3** of **Supplementary Methods**). The presence of ‘-‘ in the column ‘CTISL With marker genes’ indicates that no results are available for corresponding experiments because no overlapping marker genes in the training dataset can be used to train the model.

**Supplementary Table S7.** Cross-species cell type prediction by CTISL on human and mouse pancreas datasets

| **Training dataset** | | | **Testing dataset** | | | **Prediction results** |
| --- | --- | --- | --- | --- | --- | --- |
| Species | Cell types | Number of cells | species | Cell types | Number of cells | Number of cells accurately predicted |
| Human | T_cell | 7 | Mouse | T_cell | 7 | 0 |
|  | acinar | 958 |  | - | - | - |
|  | activated_stellate | 284 |  | activated_stellate | 14 | 11 |
|  | alpha | 2326 |  | alpha | 191 | 185 |
|  | beta | 2525 |  | beta | 894 | 852 |
|  | delta | 601 |  | delta | 218 | 76 |
|  | ductal | 1077 |  | ductal | 275 | 255 |
|  | endothelial | 252 |  | endothelial | 139 | 137 |
|  | epsilon | 18 |  | - | - | - |
|  | gamma | 255 |  | gamma | 41 | 3 |
|  | macrophage | 55 |  | macrophage | 36 | 36 |
|  | mast | 25 |  | - | - | - |
|  | quiescent_stellate | 173 |  | quiescent_stellate | 47 | 35 |
|  | schwann | 13 |  | schwann | 6 | 0 |
|  | - | - |  | B_cell | 10 | 0 |
|  | - | - |  | immuse_other | 8 | 0 |
| Mouse | T_cell | 7 | Human | T_cell | 7 | 3 |
|  | - | - |  | acinar | 958 | 0 |
|  | activated_stellate | 14 |  | activated_stellate | 284 | 229 |
|  | alpha | 191 |  | alpha | 2326 | 399 |
|  | beta | 894 |  | beta | 2525 | 2509 |
|  | delta | 218 |  | delta | 601 | 281 |
|  | ductal | 275 |  | ductal | 1077 | 1048 |
|  | endothelial | 139 |  | endothelial | 252 | 252 |
|  | - | - |  | epsilon | 18 | 0 |
|  | gamma | 41 |  | gamma | 255 | 25 |
|  | macrophage | 36 |  | macrophage | 55 | 48 |
|  | - | - |  | mast | 25 | 0 |
|  | quiescent_stellate | 47 |  | quiescent_stellate | 173 | 151 |
|  | schwann | 6 |  | schwann | 13 | 0 |
|  | B_cell | 10 |  | - | - | - |
|  | immuse_other | 8 |  | - | - | - |

Note: Several cell types (e.g., acinar, epsilon, and mast) only appear in the human dataset and are not present in the mouse dataset. Similarly, two cell types (e.g., B_cell and immuse_other) solely appear in the mouse dataset and are not present in the human dataset. Therefore, in the table, these cell types, as well as the corresponding number and accuracy of predicted cells, are indicated as '-'.

**Supplementary Table S8**. Performance evaluation details of CTISL on the 10Xv2 dataset using the five-fold cross-validation test in the intra-dataset scenario

|  | B cell | CD14+  monocyte | CD16+  monocyte | CD14+  T cell | Cytotoxic+  T cell | Dendritic  cell | Megakaryocyte | Natural killer cell | Plasmacytoid dendritic cell |
| --- | --- | --- | --- | --- | --- | --- | --- | --- | --- |
| The number of cell types | 676 | 1011 | 175 | 1456 | 2128 | 88 | 187 | 429 | 38 |
| The number of cell types accurately predicted | 671 | 997 | 169 | 1309 | 1967 | 81 | 185 | 350 | 36 |
| The percentage of cell types accurately predicted. | 99.3% | 98.6% | 96.6 | 89.9% | 92.4% | 92.0% | 98.9% | 81.6% | 94.7% |

Note: The number of accurately predicted cells is the sum of all correctly predicted cells through the five-fold cross-validation test**.**

**Supplementary Table S9**. Cell types and cell numbers in the two batches of the Retina(19) dataset

| **Retina(19)_b1** | | **Retina(19)_b2** | |
| --- | --- | --- | --- |
| **Cell types** | **Number of**  **cells** | **Cell types** | **Number of**  **cells** |
| RBC (Rod Bipolar cell) | 7065 | RBC (Rod Bipolar cell) | 3823 |
| MG (Mueller Glia) | 1219 | MG (Mueller Glia) | 1726 |
| BC5A (Cone Bipolar cell 5A) | 1074 | BC5A (Cone Bipolar cell 5A) | 1163 |
| BC6 | 855 | BC6 | 847 |
| BC7 (Cone Bipolar cell 7) | 818 | BC7 (Cone Bipolar cell 7) | 941 |
| BC5C | 487 | BC5C | 885 |
| BC1A | 486 | BC1A | 605 |
| BC3B | 350 | BC3B | 467 |
| BC1B | 340 | BC1B | 452 |
| Doublets/Contaminants | 321 | Doublets/Contaminants | 348 |
| BC3A | 239 | BC3A | 296 |
| BC2 | 196 | BC2 | 362 |
| BC5D | 156 | BC5D | 397 |
| BC4 | 118 | BC4 | 280 |
| BC8/9 (mixture of BC8 and BC9) | 108 | BC8/9 (mixture of BC8 and BC9) | 205 |
| BC5B | 72 | BC5B | 407 |
| AC (Amacrine cell) | 45 | AC (Amacrine cell) | 207 |
| Rod Photoreceptors | 29 | Rod Photoreceptors | 62 |
| Cone Photoreceptors | 9 | Cone Photoreceptors | 39 |

**Supplementary Table S10**. Cell subtypes of Retina(19)_batch1 or Retina(19)_batch2

| **Cell types/subtypes** | **Main cell types** |
| --- | --- |
| MG (Mueller Glia) | muller |
| RBC (Rod Bipolar cell) | bipolar |
| BC5A (Cone Bipolar cell 5A) |  |
| BC6 |  |
| BC7 (Cone Bipolar cell 7) |  |
| BC5C |  |
| BC1A |  |
| BC3B |  |
| BC1B |  |
| BC3A |  |
| BC2 |  |
| BC5D |  |
| BC4 |  |
| BC8/9 (mixture of BC8 and BC9 |  |
| BC5B |  |
| AC (Amacrine cell) | amacrine |
| Rod Photoreceptors | rods |
| Cone Photoreceptors | cones |
| Doublets/Contaminants | Doublets/Contaminants |


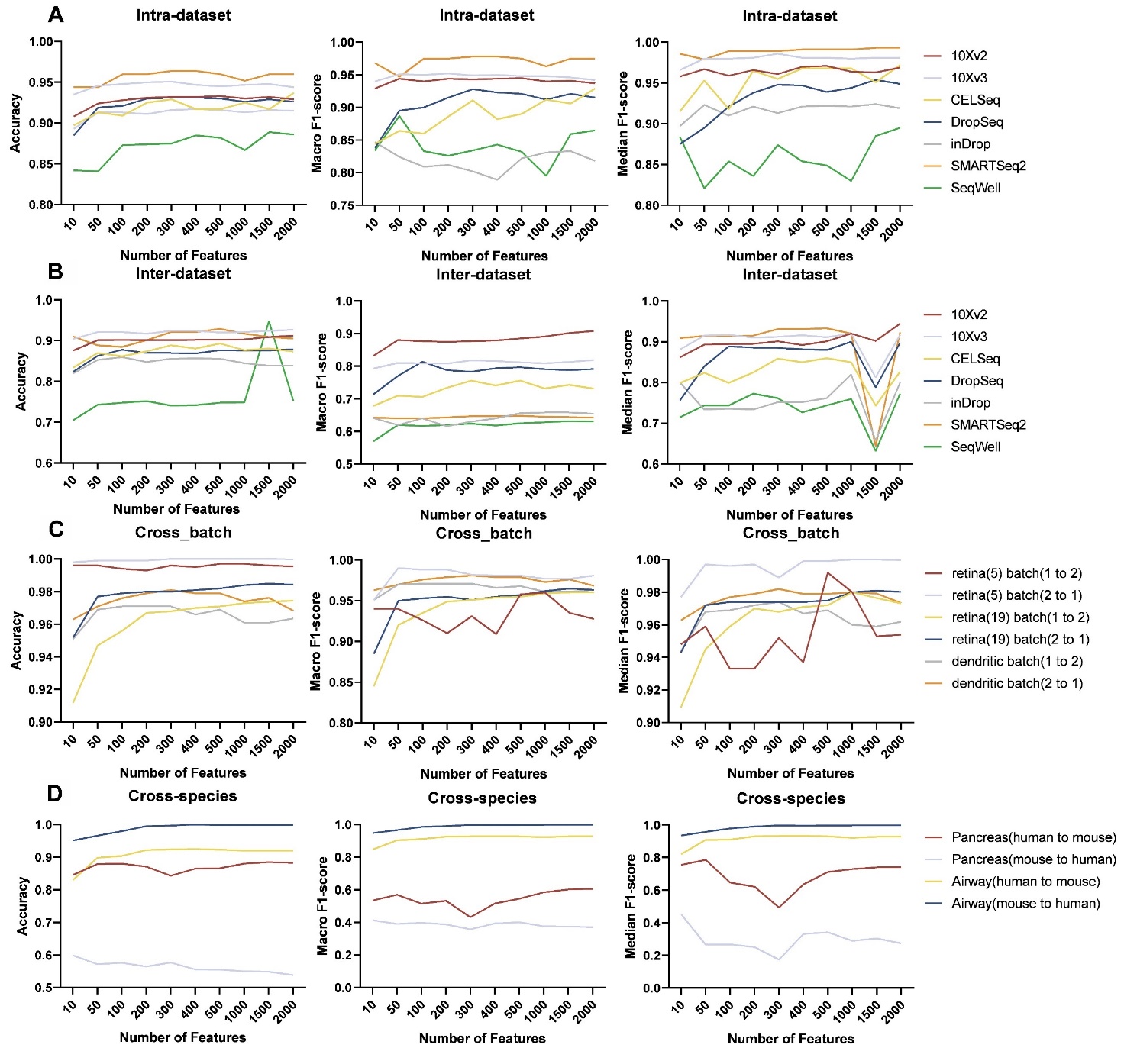


**Supplementary Figure S1.** Performance evaluation of CTISL using different numbers of selected genes by the Chi-square method in intra-dataset, inter-dataset, cross-batch dataset, and cross-species experiment scenarios.


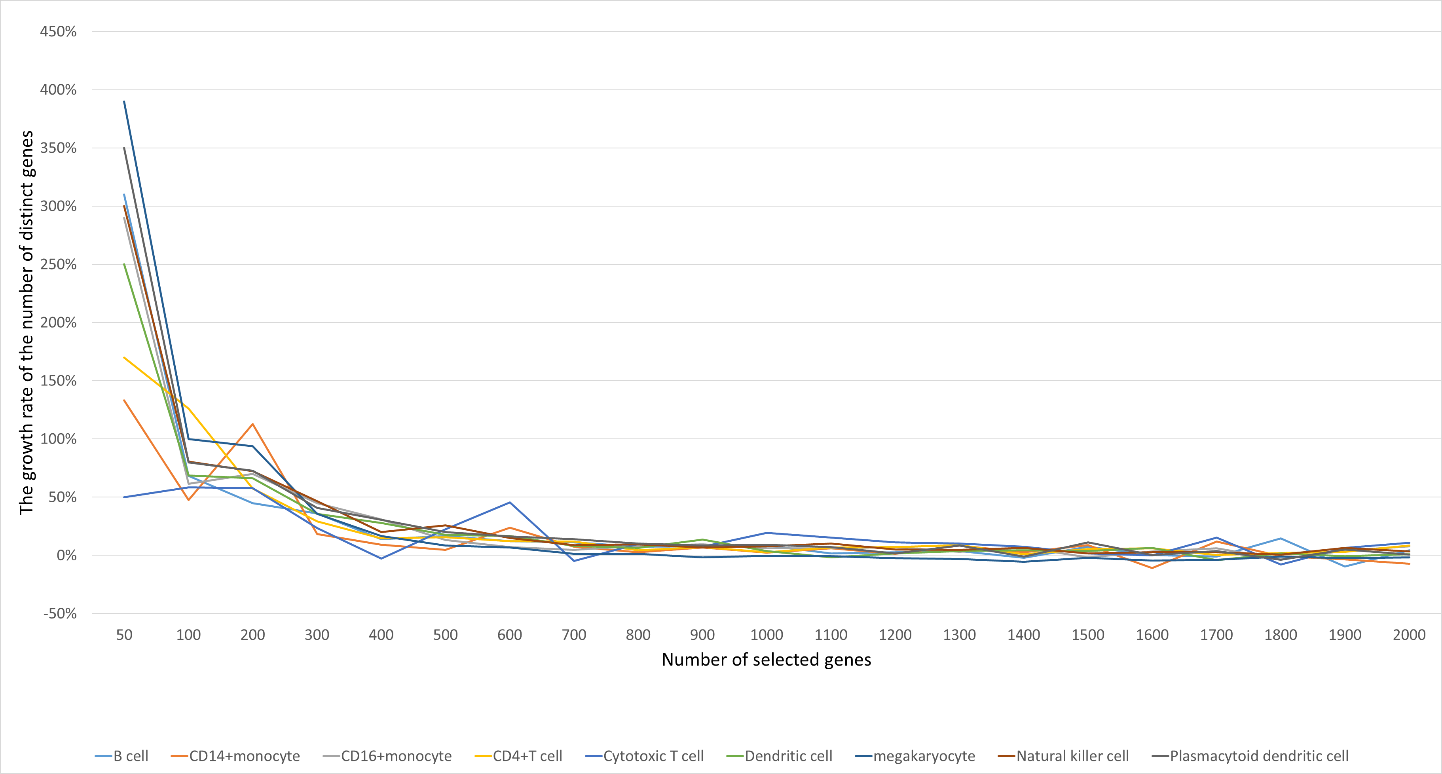


**Supplementary Figure S2**. The growth rate of the number of distinct genes for each cell type across various dimensions of feature genes selected in the 10Xv2 dataset. Suppose there are *C*_i_ distinct genes for a certain cell type on the *i*-th dimension of the x-axis. The growth rate of the number of distinct genes for the cell type is defined as (*C*_i_ - *C*_i-1_)/ *C*_i-1_ on the *i*-th dimension. Taking the B cell in the 10Xv2 dataset as an example (**Table S4**), when the number of selected feature genes was 50, there were 41 distinct genes. When the number of selected feature genes was 100, there were 69 distinct genes. In this case, the growth rate is 68.3% ((69-41)/41) when the number of selected feature genes was 100.


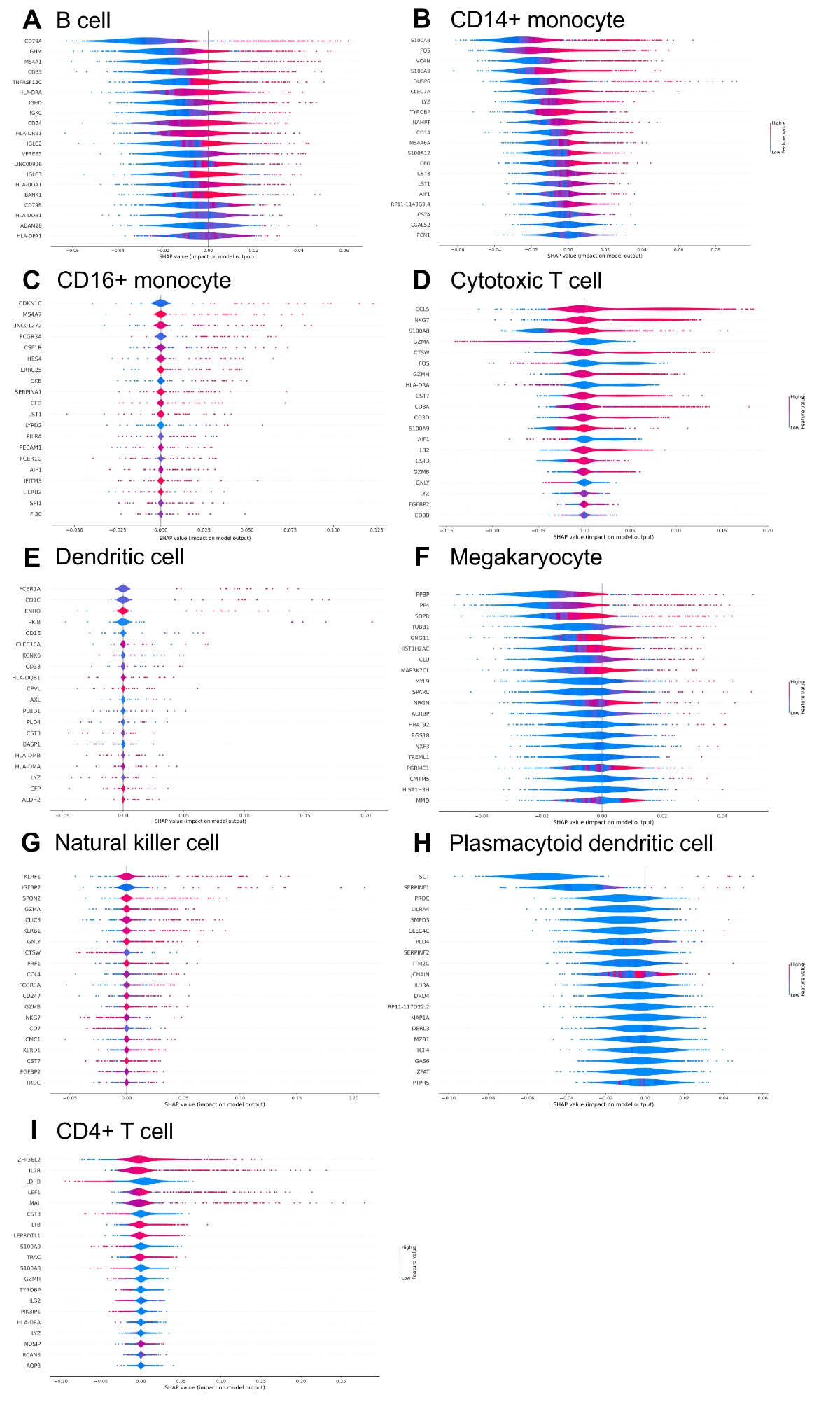


**Supplementary Figure S3**. The SHAP values of major cell types in the 10Xv2 dataset, including (A) B cell, (B) CD14+ monocyte, (C) CD16+ monocyte, (D) Cytotoxic T cell, (E) Dendritic cell, (F) Megakaryocyte, (G) Natural killer cell, (H) Plasmacytoid dendritic cell, and (I) CD4+ T cell

**
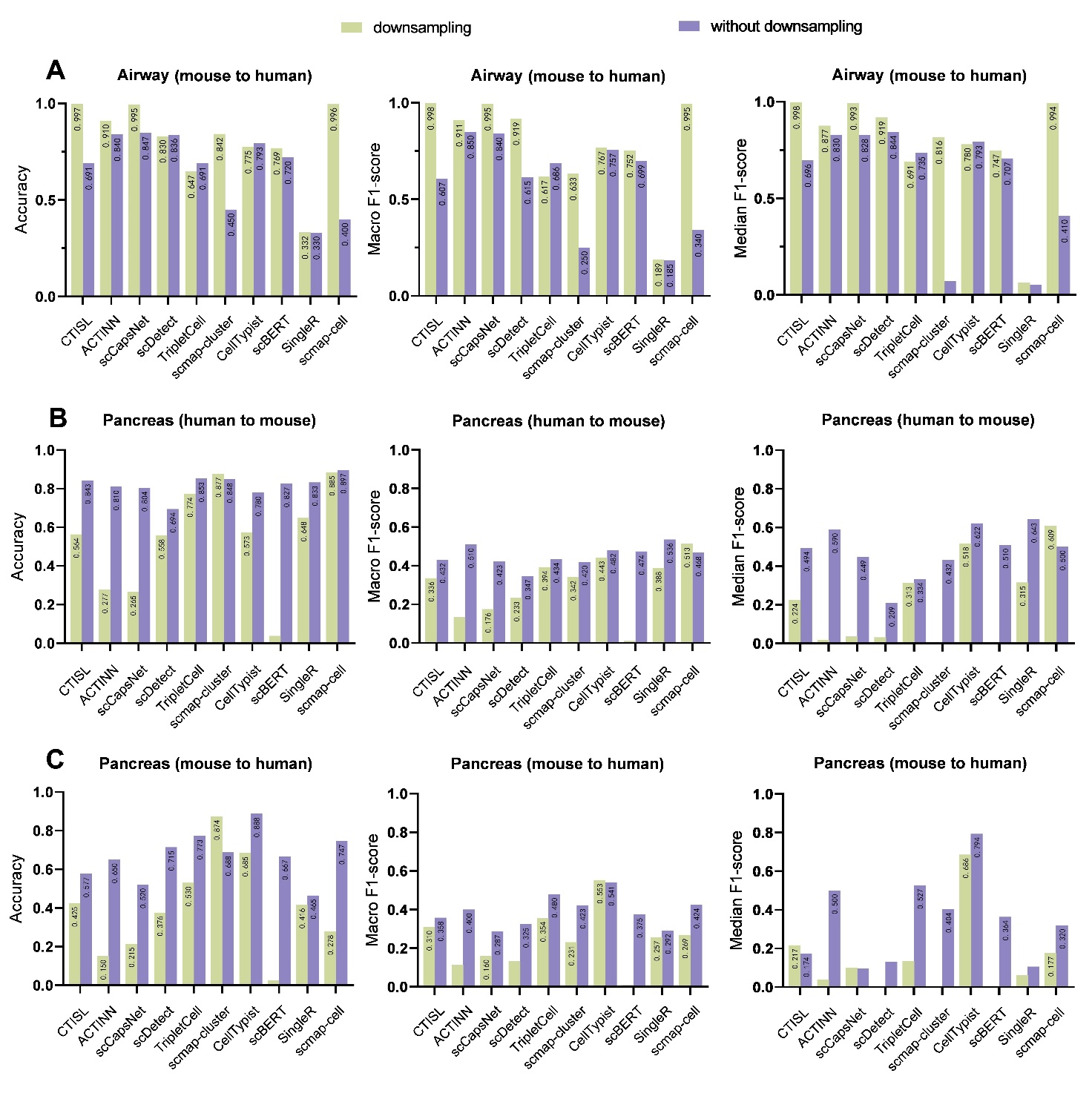
**

**Supplementary Figure S4.** Cell type prediction across species with and without down-sampling using CTISL. (A) the model trained on the mouse airway dataset was used to predict cell types in the human airway dataset. (B) The model trained on the human pancreas dataset was used to predict cell type in the mouse pancreas dataset. (C) the model trained on the mouse pancreas dataset was used to predict cell type in the human pancreas dataset.

References

Baron, M.*, et al.* A Single-Cell Transcriptomic Map of the Human and Mouse Pancreas Reveals Inter- and Intra-cell Population Structure. *Cell Systems* 2016;3(4):346-+.

Breiman, L. Random Forests. *Machine Learning* 2001;45(1):5-32.

Friedman, J.H. Greedy Function Approximation: A Gradient Boosting Machine. *The Annals of Statistics* 2001;29(5):1189-1232.

Hu, C.*, et al.* CellMarker 2.0: an updated database of manually curated cell markers in human/mouse and web tools based on scRNA-seq data. *Nucleic Acids Research* 2023;51(D1):D870-D876.

Laurikkala, J. Improving identification of difficult small classes by balancing class distribution. In, *Artificial Intelligence in Medicine: 8th Conference on Artificial Intelligence in Medicine in Europe, AIME 2001 Cascais, Portugal, July 1–4, 2001, Proceedings 8*. Springer; 2001. p. 63-66.

Lemaître, G., Nogueira, F. and Aridas, C.K.J.T.J.o.M.L.R. Imbalanced-learn: A python toolbox to tackle the curse of imbalanced datasets in machine learning. *Journal of Machine Learning Research* 2017;18(1):559-563.

Popescu, M.-C.*, et al.* Multilayer perceptron and neural networks. *WSEAS Transactions on Circuits and Systems* 2009;8(7):579-588.

Shekhar, K.*, et al.* Comprehensive Classification of Retinal Bipolar Neurons by Single-Cell Transcriptomics. *Cell* 2016;166(5):1308-1323.e1330.

Villani, A.-C.*, et al.* Single-cell RNA-seq reveals new types of human blood dendritic cells, monocytes, and progenitors. *Science* 2017;356(6335):eaah4573.
